# Latent influence networks in global environmental politics

**DOI:** 10.1371/journal.pone.0213284

**Published:** 2019-03-07

**Authors:** Benjamin W. Campbell, Frank W. Marrs, Tobias Böhmelt, Bailey K. Fosdick, Skyler J. Cranmer

**Affiliations:** 1 Department of Political Science, The Ohio State University, Columbus, OH, United States of America; 2 Department of Statistics, Colorado State University, Fort Collins, CO, United States of America; 3 Department of Government, University of Essex, Colchester, United Kingdom; Institut Català de Paleoecologia Humana i Evolució Social (IPHES), SPAIN

## Abstract

International environmental treaties are the key means by which states overcome collective action problems and make specific commitments to address environmental issues. However, systematically assessing states’ influence in promoting global environmental protection has proven difficult. Analyzing newly compiled data with a purpose-built statistical model, we provide a novel measurement of state influence within the scope of environmental politics and find strong influences among states and treaties. Specifically, we report evidence that states are less likely to ratify when states within their region ratify, and results suggesting that countries positively influence other countries at similar levels of economic development. By examining several prominent treaties, we illustrate the complex nature of influence: a single act of ratification can dramatically reshape global environmental politics. More generally, our findings and approach provide an innovative means to understand the evolution and complexity of international environmental protection.

## Introduction

The international dynamics underlying interstate environmental politics are increasingly important to understand because effective international environmental policy, as is the case with other areas of international policy, depends upon the concerted efforts of national governments and is prone to a litany of collective action problems. The most public means by which states can pledge to take environmental action is by ratifying, joining, or acceding international environmental treaties, particularly those with detailed and enforceable provisions about actions to be taken. Joining international environmental agreements via ratification comprises benefits, particularly in the long run as more concerted efforts toward solving environmental problems become possible and environmental quality may improve due to these, but also costs as actors have to somewhat alter existing patterns of behavior in order to overcome inefficient or ineffective policies. Importantly, ratification decisions are not made in a vacuum and are often thought to be linked to the actions of other states. For instance, consider the problem of air pollution and acid deposition (acid rain) in Europe in the 1970s. While initial efforts to address this issue internationally failed, due to pressures from the US and the Eastern bloc during the Cold War, it was primarily the influence of the Nordic states and Germany that eventually led to the establishment of the Convention on Long-Range Transboundary Air Pollution in 1979 and other states joining this agreement [1, 2]. This illustration underlines that actor linkages [3, 4] and structural group leadership [5] are frequently seen as key determinants in international environmental governance. And, indeed, more recently have EU states been aspiring to leadership roles in global environmental policymaking and to influence others to join their efforts. To analyze these foundational dynamics underlying international environmental politics, we developed a novel method to estimate the international influence network and how it informs one of the most pressing issues of our time—environmental protection.

International influence—the pressure exerted by one state to get another to do something it would not otherwise do—has often been touted as a means by which to compel coordinated action [5, 6]. Consider the canonical collective action problem and its relevance to the subject of environmental protection—preservation of the environment requires that states cooperate and commit to behavioral changes to provide the public good of environmental quality. Within this dynamic, we might expect one state’s action to either promote the action of another or promote the inaction of another as incentives for inaction mount, allowing such a state to, e.g., benefit from economic gains due to laxer environmental standards. However, the fact remains that we “know relatively little about the conditions under which such attempts are likely to succeed,” [5]. Indeed, state influence is a foundational concept for scholars and practitioners of international politics [7, 8], and while there have been attempts to develop systematic measurements of influence [9, 10], such approaches suffer from interpretation and inferential challenges [11].

States can commit to cooperative efforts via the ratification of or accession to a formal treaty that addresses an international (environmental) problem. An accurate understanding of this “ratification phenomenon” is not only relevant for scholars, but also for policymakers and public institutions. Existing studies focus on three types of determinants of states’ participation in international agreements that may pertain to either costs or benefits associated with the ratification decision: (1) treaty design characteristics, (2) domestic influences, and (3) international systemic factors [12–16]. First, international agreements vary considerably in their design. These institutions can be highly legalized if they are, for example, characterized by clear obligations (i.e., an institution’s rules can be enforced upon its members), are precise (i.e., rules are clearly and unambiguously defined), and delegate authority to a supranational body (i.e., a body like a secretariat that has authority to implement, interpret, and apply rules). In general, states are more reluctant to ratify more legalized agreements [17]. Second, there are domestic-level characteristics such as political regime type, institutional constraints, or countries’ economic conditions. It is argued, for instance, that democracies are more likely than non-democratic regimes to provide public goods and, thus, are also more inclined to cooperate in international environmental problem-solving efforts. Third, there are systemic factors. For example, other agreements such as preferential trade agreements may affect treaty ratification and there is likely a contingent behavior of countries, i.e., a state’s degree of cooperative behavior is influenced by other nations’ actions. However, while policymakers and diplomats have long claimed that states influence each other in their decisions at the international level, including ratification patterns [18–20], systematically assessing and estimating this influence has proven difficult. Previous work largely neglects state-state influences, treaty-treaty effects, and impacts that diffuse via treaty-state links; and existing evidence of state-state influence is largely based on single cases or produced inconclusive results.

Our premise is that both states and treaties form a network, which creates interdependencies *between* states and treaties as well as *among* states and treaties, both of which influence the likelihood of ratification (i.e., joining an agreement). Our interest here is in the latter. However, not all states or treaties in these networks are created equal. We concentrate on relative positions, characteristics, and importance in the network for assessing which states are more likely to push others to ratify certain treaties. Because we seek to account for temporal dynamics, along with properties of the network structure, we begin by introducing newly expanded data that are based on earlier coding efforts [14, 19] of state ratifications of international environmental agreements. The challenge of modeling international influence is complicated by the structure in which states’ ties to treaties affect subsequent ties. The recently introduced Bipartite Longitudinal Influence Network (BLIN) model [11] accounts for these network dependencies among two sets of actors, resulting in a flexible framework for complex data, which is useful for inference, prediction, and interpretation. It models the evolution of state actions using a temporal process that can also accommodate actor and relationship-level predictors typical of the literature [12–16]. Building on earlier work, [21–23], these methods allow for inferring latent network structure based on temporal bipartite event data, which models the data directly rather than a transformation of the data that would lead to a reduction in informational content [17].

We address three crucial, yet unanswered questions. Which states influence others’ ratification behavior? What is the importance of the state influence and treaty-issue linkage for states’ ratification behavior? And how is ratification determined by the joint impact of national attributes, network dependencies, and spatial effects? Answering these questions has implications for our understanding of global environmental governance, state-to-state influence, and nations’ ratification behavior in the international system. Uncovering the international-influence network presents an opportunity to understand the nature of state influence broadly, and, within this particular context, to shed light on the collective action problems that characterize international environmental protection. It also provides an opportunity to understand how treaties may influence other agreements and, ultimately, reveal the previously unmeasured state-to-state influence network.

## Design

To assess the proposition that influence networks help shape the evolution of international environmental protection, we augment and analyze data on international environmental treaty ratification. We compiled data on international environmental agreements, which focus on treaties that were open for ratification globally between 1972 and 2000. We based the coding of the data on the original treaty documents and texts, which do hardly change over time. With this re-coding of the ratification data for every state-treaty pair, we not only validate the original data from [19], but add information on the exact day and month of a ratification event. Second, we updated the data on available covariates, doing much to address the problem of missing values in the original data. The outcome variable for our models is a binary indicator for whether a state ratified a given treaty in the year of observation (see Methods for details). The inclusion of a state-treaty-year triplet in the data is an indication that a state could have ratified a treaty during the given year. In other words, for a state-treaty-year to be included, the state has to have had the potential to ratify the treaty in that given year. A state-treaty pair remains in the data until 2000 if no ratification occurs, but leaves the data before if it has joined an agreement.

The analysis of these data is complicated by the fact that more than the interactions between states and treaties, relationships, and network topology are at work: the temporal dynamics involved in network evolution are generally substantial and must be accounted for both theoretically and empirically. The estimation challenge is made more difficult by the fact that few techniques exist to model highly dynamic bipartite data—data such as ours where the network consists of two different types of nodes whose relations evolve over time. Because of these difficulties, researchers often aggregate network data over time and project bipartite networks to create a single binary unipartite static network [24]. This aggregation causes a significant loss of information contained in the temporal dynamics and the projection from the bipartite network to the unipartite network makes strong assumptions about dependencies between actors that are not well understood, while discarding a substantial portion of the information content in the data. Some existing methods for analyzing bipartite networks that evolve over time may allow analysts to assess the structure of a network, but do not allow for the inference of influence networks [25, 26]. Others allow for the inference of influence networks, but do not disentangle within-mode influence [9, 10] and, hence, face interpretation and inferential challenges [11]. To leverage the information inherent in temporal bipartite event data while inferring latent influence network structure, we utilize the recently introduced BLIN model which models the data generating process directly [11].

The BLIN model accounts for the influence relationships of interest in a generative manner. This model is advantageous over previously discussed work in that it is parsimonious, interpretable, and flexible in its ability to capture network-based dependencies among both sets of node entities in the bipartite network (see [11] and S1 Appendix for additional details on the BLIN model). A key advantage of the BLIN model is that the influence networks may be estimated and their uncertainty assessed using existing software tools for linear models.

### Model validation and comparison to the state of the art

To infer the joint effect of influence networks and covariates on environmental treaty ratification, we specify a BLIN model for all environmental treaties and states from 1972 to 2000. Given that the process of treaty ratification might span more than one year and, thus, can be gradual, we allow for the possibility of state and treaty influence to occur over a three-year period. In addition, we include a series of covariates conventionally considered when predicting environmental treaty ratification [17, 19, 20, 27]. These predictors are discussed in the S1 Appendix and their estimated effects are presented in Table 1.

**Table 1:** BLIN and penalized logit results for environmental treaty ratification. A Lasso regularization parameter which maximized AUCPR in 10-fold cross-validation was used for each model. The use of the BLIN model over penalized logit improves the predictive performance of the model: AUCPR increases by 0.6 when using BLIN over penalized logit. The substantive implications of these models differ in interpreting control variables. Hard law, legislative approval, log SO_2_, log GDP per capita, global mixed goods, and log economic openness are all regularized to zero when including network effects. Significance was determined using the selectiveInference package, which returns a *p*-value for each coefficient. We indicate significance of a given coefficient at the 0.05 level.

	Logit Model	BLIN Model
Intercept	−4.55* (0.00)	−5.80* (0.00)
Polity	0.02* (0.00)	0.01 (0.00)
Hard Law	−0.15* (0.03)	0.00
Legislative Approval	0.00 (0.04)	0.00
ln SO_2_ PC	0.04* (0.01)	0.00
ln GDP per capita	−0.09* (0.02)	0.00
Assistance	0.49* (0.04)	0.13 (0.07)
Assistance to Developing States	0.85* (0.05)	0.27 (0.09)
Global Public Good	−0.40* (0.03)	−0.17 (0.06)
Global Mixed Good	−0.14 (0.07)	0.00
ln Openness	0.11* (0.02)	0.00
IO Memberships	0.02* (0.00)	0.01 (0.00)
Lag States Ratifying	0.01* (0.00)	0.00
Lag States in Region Ratifying	0.03* (0.00)	0.03 (0.00)
Lag States in Income Group Ratifying	0.02* (0.00)	0.00 (0.00)
ln GDP	0.12* (0.01)	0.01 (0.00)
*t*	−0.27* (0.01)	−0.08 (0.01)
*t*^2^	0.01* (0.00)	0.00
*t*^3^	0.00* (0.00)	0.00 (0.00)
AUCPR	0.10	0.70

Note:

* and bolding indicate significant at the 0.05 level.

We validate our model by examining the substantive changes that occur when comparing our model that accounts for network dependencies to a state-of-the-art model in the literature, which we replicate in Table 1 using penalized logistic regression [20]. We note that the BLIN model explains significantly more of the within-sample variation in ratifications as demonstrated by the AUCPR = 0.70 (area under the precision recall curve) compared to AUCPR = 0.10 for the logistic regression. In addition, we find that the BLIN model predicts future ratifications at least as well as the comparison model, as measured by out-of-sample AUCPR, when fitting to consecutive 10-year windows of the data (for more details, see the S1 Appendix). In other words, our model predicts as well or better than the state-of-the art-model, while explaining the variation of the data within sample much more accurately. This suggests that the BLIN model, rather than overfitting, is explaining systematic network effects of environmental treaty ratification.

When accounting for network effects using the BLIN model, the evidence for some of the variables conventionally considered to affect the likelihood of treaty ratification becomes somewhat ambiguous. For instance, we no longer find support for a substantive influence of the number of states from the same income group that have ratified a particular treaty. This is not surprising, however, as the BLIN estimation incorporates modeling the network structure directly, which is likely to confound that variable. The degree of legalization and some state attributes such as sulfur dioxide emissions (used as a proxy for environmental quality), income, or economic openness are also no longer associated with a substantive influence [19, 20]. In summary, we shed new light on network interdependencies, which we explicitly consider and model. To this end, the most innovative aspect of the BLIN model is that we now can assess the influence that specific states exercise over other states, and how the ratification of one treaty may promote or discourage the ratification of another. The core of our investigation is, therefore, the analysis of the state influence network, which we present in the following sections (for robustness checks and a discussion of accounting for particular international organizations, see the S1 Appendix).

## Results

From patterns in environmental treaty ratification, the BLIN infers two weighted, directed networks. These networks describe either the positive or negative influence among states and treaties. Negative influence occurs when the one event makes another potential event less likely. For example, if Ireland ratifies a given treaty, France may be less likely to ratify the same treaty. Positive influence reverses this dynamic, wherein one event makes another potential event more likely. For example, if Iceland ratifies a given treaty, Norway may be more likely to ratify the same. These influence relationships are also weighted such that larger weights refer to stronger influence relationships between actors. To be clear, the BLIN model provides correlational results that are consistent with the causal process of influence. With observational network data such as ours, a direct causal test (e.g., an experiment or matching process that relies on independent observations) is not possible. For ease of interpretation, we parse the positive and negative influence networks into four different networks based upon the direction of influence and the nodes included.

### Negative state influence network

Within the context of public (and also non-excludable mixed) goods provision, the phenomena of free-riding is common [28]. Environmental protection or quality is usually seen as a public good that may be sufficiently provided by the cooperative efforts of a set of states. Free-riding is thought to occur when an actor can, through inaction, access a public good provided by others without contributing to the costs of action and deriving benefits in other areas that may be affected by more stringent environmental regulations. As such, some states may be able to benefit from better environmental quality or economic advantages without incurring some or all of the costs stemming from treaty ratification. While we may not observe free-riding in its truest sense, we observe a form of “negative influence” wherein the action of some countries makes others less likely to act [5, 6]. In other words, negative influence occurs when one state ratifying a particular treaty means that another state is less likely to do the same with that particular agreement. However, we are hesitant to treat negative influence and free-riding as complete synonyms as there is a variety of other factors that must be considered, such as whether the treaty has been enacted or if phenomena other than free-riding are consistent with negative influence.

**Fig 1:**
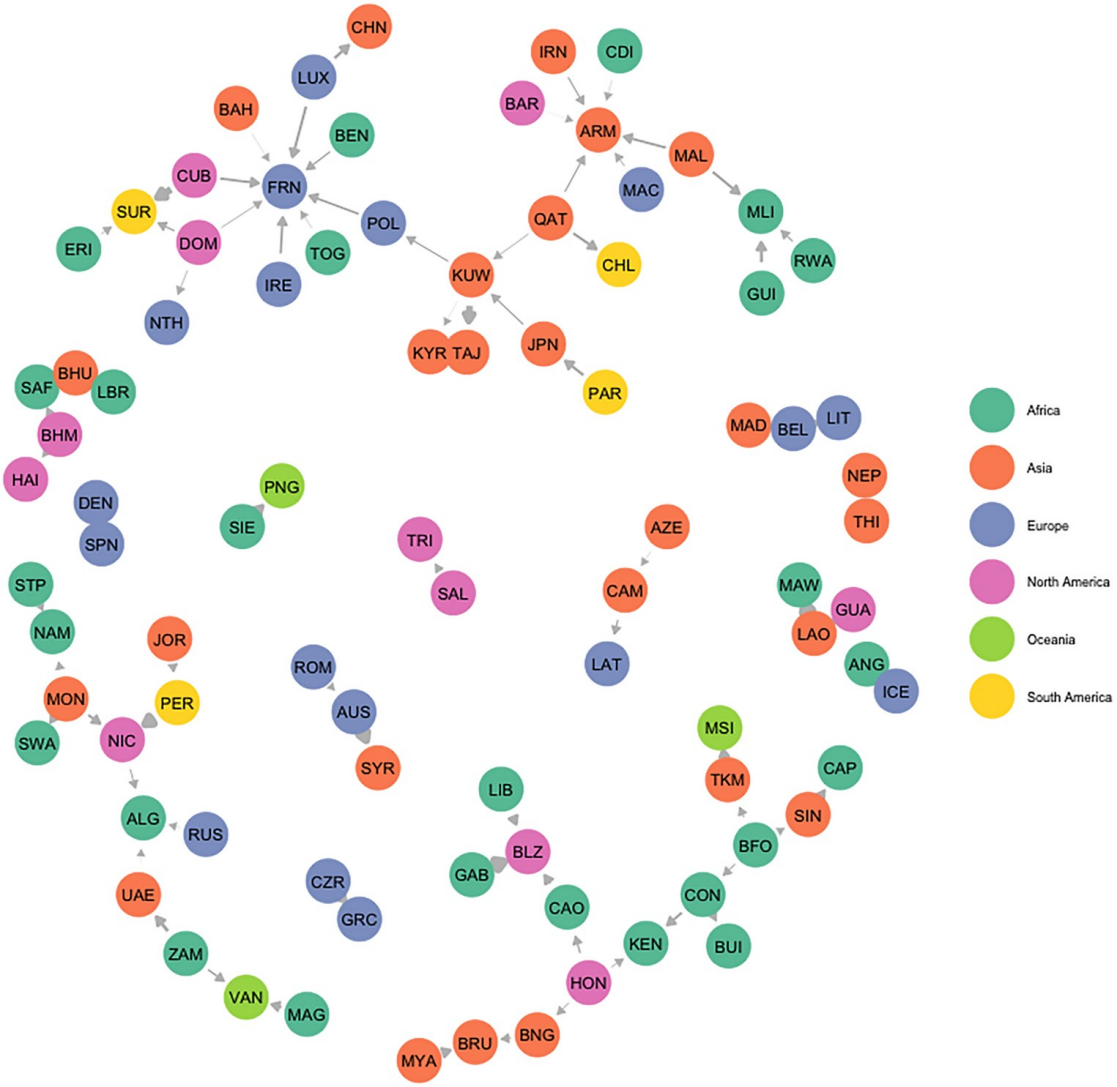
Negative state influence network. All edges represent negative influence parameters significant at the 0.05 level. Nodes are labeled according to the Correlates of War alpha-3 codes and colored according to region. The size of the edges is proportional to the relative degree of influence the sender exercises over the receiver. One of the strongest negative influence relationships is between Kuwait and Tajikistan, which implies that should the former ratify a treaty, the latter would be less likely to ratify the same treaty. This network is fairly sparse, with few states being negatively influenced by many more than one or two states.

The negative state influence network estimated by the BLIN model (Fig 1) demonstrates that those not ratifying when a negative influencer ratifies are less likely to be induced to inaction by democracies and more likely to be induced to inaction by relatively few states and states within the same continent. In this context, one of the strongest negative influence relationships is between Kuwait and Tajikistan, which implies that should the former ratify a treaty, the latter would be less likely to ratify the same treaty. More intuitively perhaps, consider Qatar and Kuwait, which are both major oil-exporting countries and members of the Organization of the Petroleum Exporting Countries (OPEC) until 2019. Qatar left OPEC on January 1, 2019, while Kuwait remained a member of that organization, mirroring our finding that when Qatar ratifies an environmental agreement treaty, Kuwait would be less likely to ratify the same. To further examine these patterns, we examine the negative influence network via an Exponential Random Graph Model (ERGM) estimated through Markov Chain Maximum Likelihood Estimation (MCMLE) [29, 30]. The ERGM results (Table 2) confirm two expected dynamics informing the structure of the negative influence network. On one hand, this network is particularly sparse, i.e., there are relatively few cases of negative influence for the total number of states in the international system. This demonstrates that, although present, negative influence is fairly uncommon, at least in the case of environmental treaty ratification. When states are induced to inaction, they make their decision based upon the actions of relatively few states, as supported by the negative effect for the in-degree term. This is an interesting development as most political economists do not have strong *a priori* expectations about the prevalence of negative influence, and may, on average, actually expect negative influence to be particularly common in the environmental action sphere which mirrors the tragedy of the commons problem [28]. On the other hand, states are less likely to be induced to inaction by democracies. This is an interesting dynamic that may indicate the leadership of democratic states in the current international order [31]. Finally, as expected, states are less likely to ratify treaties when states within their continent have ratified the treaty. This result pushes back against the claim that attempts to strengthen regional integration have succeeded and, in fact, may facilitate collective action. Instead, it indicates that there may be regional economic competition and concerns about relative gains or losses when states assess make decisions regarding environmental treaties.

**Table 2:** ERGM results for assessing influence network structure. Models reflect the best fitting ERGM found for each network, diagnostics indicate convergence of Markov chains. Geometric weights (GW) of 0.1, reflecting a moderate degree of down-weighting, were used for all GW terms including Geometrically Weighted In-Degree (GW In-Degree) or Out-Degree (GW Out-Degree). ERGM model results illustrate the structural features of each network. Negative State Influence: This network is constituted by relatively few negative influencees (negative and robust effect for GW In-Degree) and constituted by relatively few instances of being induced to inaction by democracies (negative and robust effect for Sender Regime Score). Positive State Influence: This network is marked by states influencing others of similar capabilities (negative and robust effect for dyadic GDP difference variable). In addition, those who are positively influenced are influenced by few states (negative and robust effect for GW In-Degree). Alternatively, those that influence others, are unlikely to influence many (negative and robust effect for GW Out-Degree Term) Negative Treaty Influence: Network has no real discernible features. Positive Treaty Influence: Network has no real discernible features. Should treaties positively influence other treaties, they are likely to influence few treaties (negative and robust effect for GW Out-Degree term). Should treaties be positively influenced by other treaties, they are likely to be influenced by few treaties (negative and robust effect for GW In-Degree).

	State, Negative	State, Positive	Treaty, Negative	Treaty, Positive
Edges	−6.49* (1.30)	−4.40* (0.79)	−8.11* (0.42)	−4.10* (0.54)
Continent Homophily	0.86* (0.24)	0.38 (0.21)		
Receiver GDP	0.12 (0.10)	−0.01 (0.08)		
Sender GOP	0.05 (0.12)	0.05 (0.09)		
Receiver Regime Score	0.01 (0.02)			
Sender Regime Score	−0.05* (0.02)			
GW In-Degree (0.1)	−2.00* (0.42)	−1.36* (0.34)		−3.31* (0.47)
GW Out-Degree (0.1)	−0.04 (0.48)	−0.74* (0.36)		−1.28* (0.52)
Isolates	0.22 (0.37)	0.44 (0.31)		
GDP Difference		−0.43* (0.13)		
Hard Law Difference			0.25 (0.50)	
Subject Homophily			−0.04 (0.54)	0.08 (0.32)
Mixing: Hard Law to Soft Law				−0.42 (0.53)
Mixing: Soft Law to Hard Law				−0.13 (0.41)
Mixing: Hard Law to Hard Law				0.32 (0.34)
AIC	1020.35	1457.07	293.48	546.79
BIC	1096.84	1525.06	319.78	609.25
Log Likelihood	-501.17	-720.53	-143.74	-266.40

Note:

* and bolding indicate significant at the 0.05 level.

### Positive state influence network

To compel action for environmental protection, states may exercise influence to promote treaty ratification. Power and influence are tools that actors may use to overcome a collective action problem [5, 6, 32]. This is a form of power that is conventionally considered by sociologists and political scientists, wherein some actor uses their power to influence another actor to do something they would not otherwise do [8, 33–35]. In the context of environmental treaties, states influence other countries to ratify an environmental treaty that they might not otherwise join. We refer to this form of influence as “positive influence.” Positive influence occurs when one state’s ratification of a given environmental treaty makes another state more likely to join the same.

**Fig 2:**
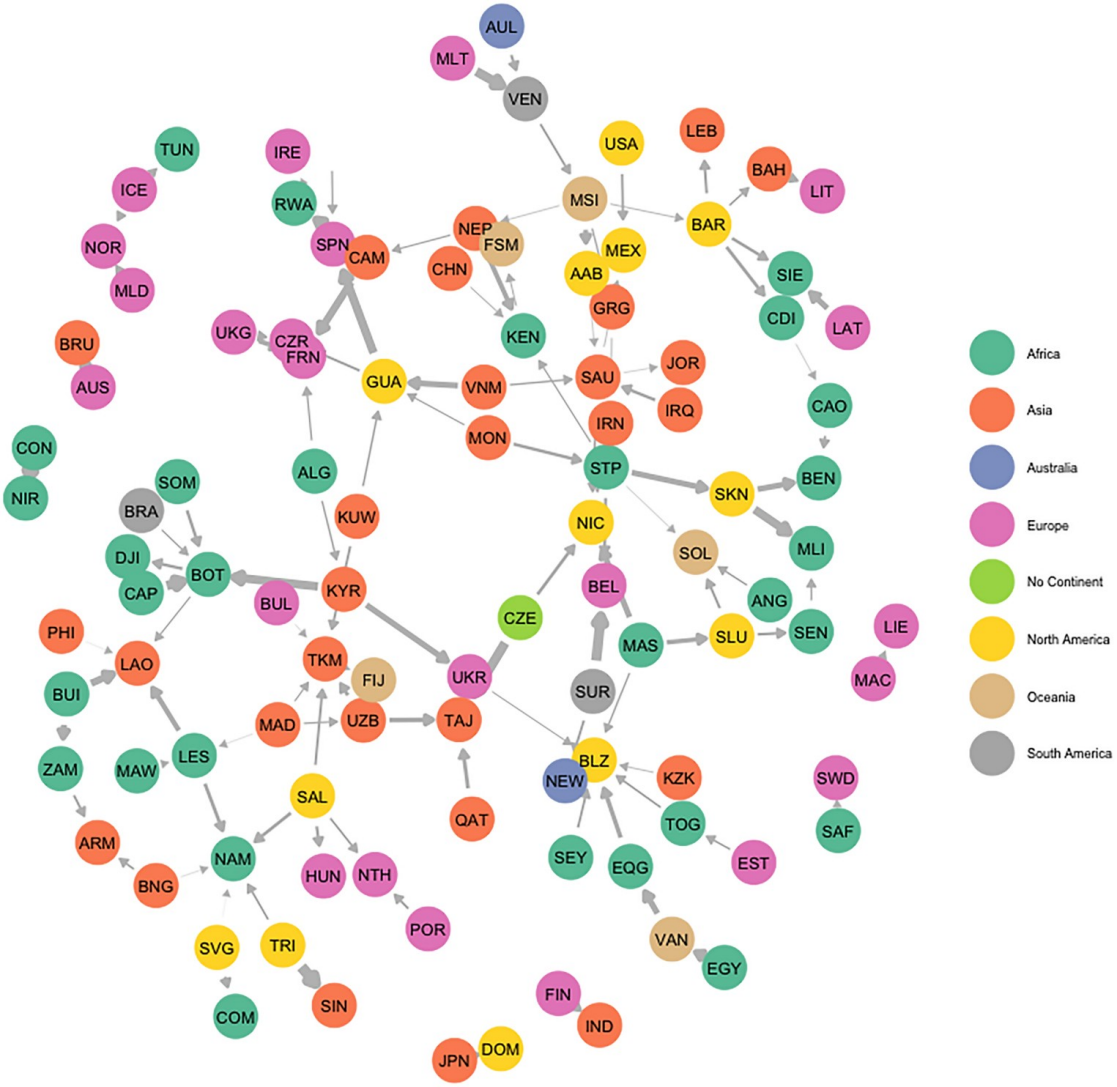
Positive state influence network. All edges represent positive influence parameters significant at the 0.05 level. Nodes are labeled according to their Correlates of War alpha-3 codes and colored according to region. The size of the edges is proportional to the relative degree of influence the sender exercises over the receiver. On of the strongest positive influence relationships is between Cape Verde and Botswana, indicating that should Cape Verde ratify a given environmental treaty, Bostwana is more likely to ratify the same treaty.

The positive state influence network (Fig 2) reflects a process of states following others in treaty ratification. It is informed by a tendency of states to be influenced by relatively few states and to influence states of similar levels of economic productivity. On of the strongest positive influence relationships is between Cape Verde and Botswana, indicating that should Cape Verde ratify a given environmental treaty, Bostwana is more likely to ratify the same treaty. Cape Verde is a small island nation, also part of the Alliance of Small Island States, and particularly vulnerable to the impact of climate change; their ratification efforts may then signal to others the need and necessity of joining and committing to an international problem-solving effort. In a sense, Cape Verde represents an opinion leader. Other positive relationships include the UK to France and Iceland to Norway—both dyads pertaining to European states that have otherwise close cooperative ties to begin with. Finally, US ratification efforts seem to strongly positively influence whether Mexico joins the same treaty. The ERGM estimated on this network (Table 2) confirms our expectation that the positive state influence network is sparse. Within the scope of environmental issues, few states actually appear to exercise influence over others. But when influence occurs, we find that it is likely to emerge between states that are relatively similar with respect to economic productivity. Interestingly, earlier work finds that economically stronger states exercise influence over weaker states [36]. We do not find evidence for region homophily within this network, which is counterintuitive as one would expect a state to exercise influence over another within close geographical proximity when positive influence exists.

### Negative treaty influence network

**Fig 3:**
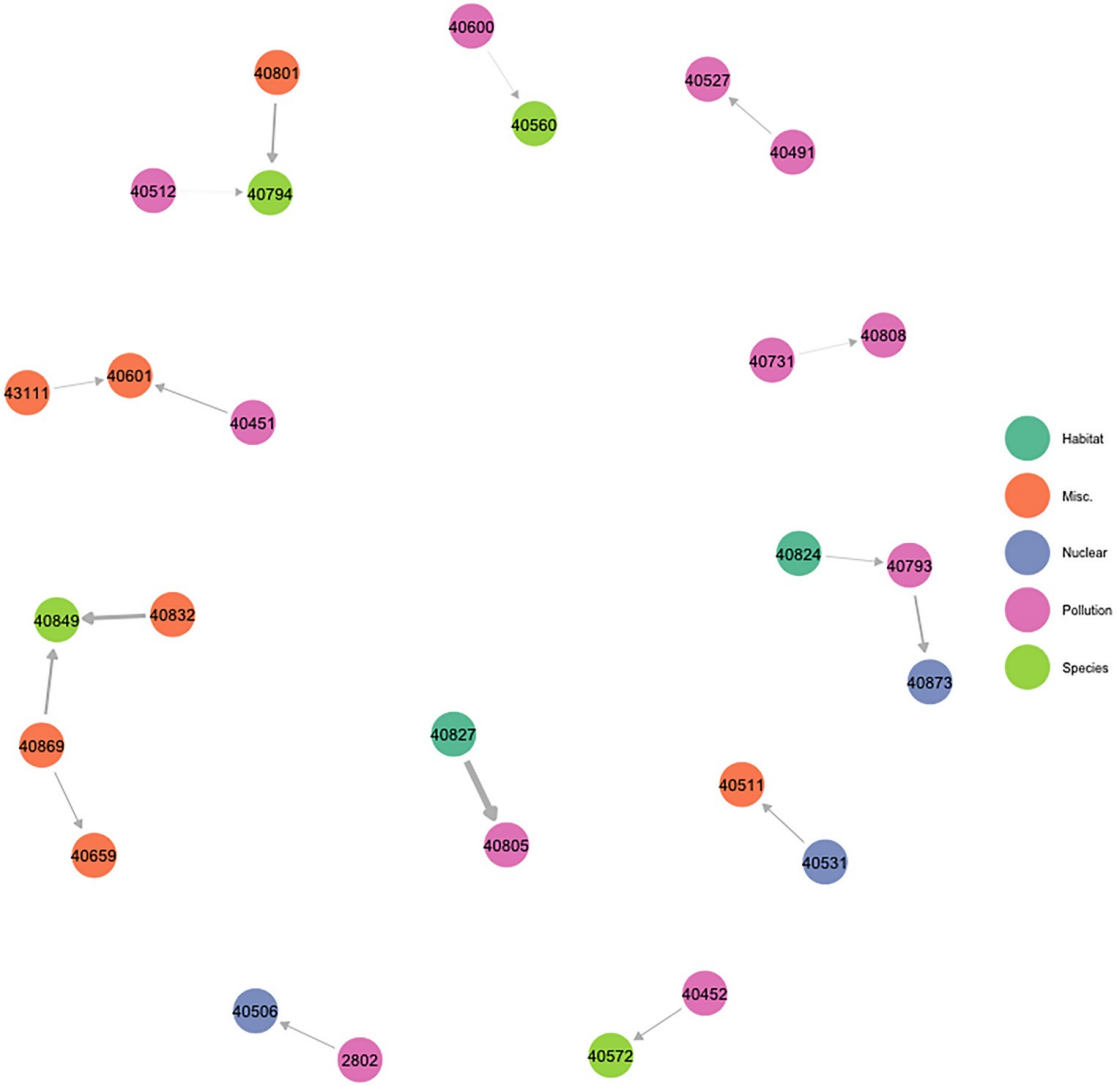
Negative treaty influence network. All edges represent negative influence parameters significant at the 0.05 level. Nodes are labeled according to their treaty numbers and colored according to topic. The S1 Appendix contains a key linking treaty ID numbers to their corresponding names. The size of the edges is proportional to the relative degree of influence the sender exercises over the receiver. One of the strongest relationships is between the United Nations Convention To Combat Desertification (treaty #40827) and the Regional Agreement on the Transboundary Movement of Hazardous Wastes (treaty #40805), implying that a state’s ratification of the former makes ratification of the latter less likely.

Treaties may occasionally be competitive. One treaty could replace another, be an alternative mechanism to accomplish the same goal, or even have incompatible targets. Similar to the state case, negative influence may also occur when examining treaty ratifications. The negative treaty influence network uncovers potential competition among treaties, wherein a state’s ratification of a particular treaty means they are less likely to ratify another agreement. The treaty negative influence network uncovered by the BLIN model is presented in Fig 3, which illustrates that there appears to be relatively little consistent structure for this network.

An ERGM fit on this network reveals that it is generated by a tendency of agreements to be negatively influenced by few other treaties. Overall, there are relatively few instances of negative treaty influence, and within the context of treaties, few ratifications preclude others. However, this may not be necessarily surprising given that we focus on one issue area only—environmental politics. This result could change drastically when incorporating additional issue areas into the data.

### Positive treaty influence network

**Fig 4:**
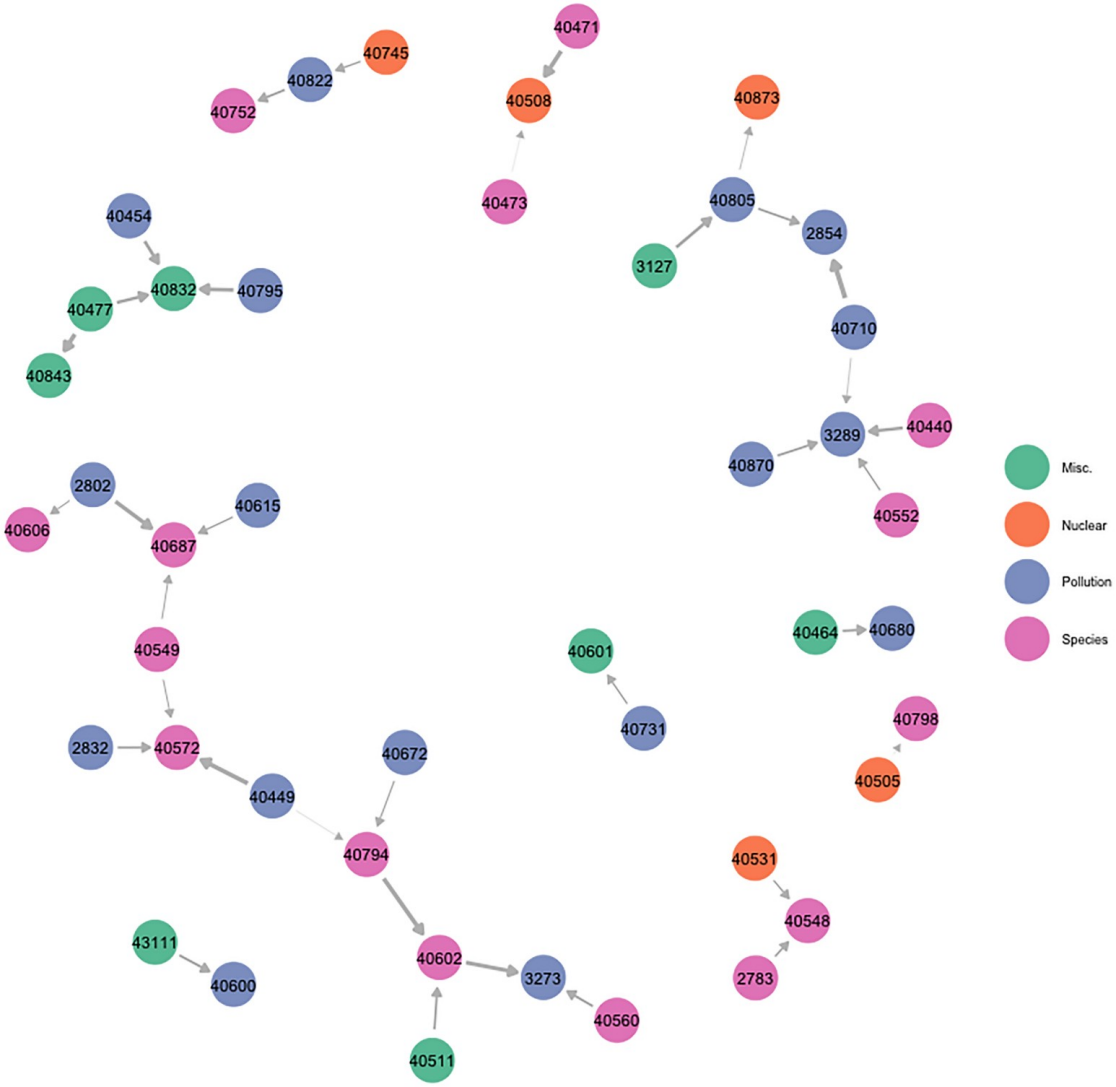
Positive treaty influence network. All edges represent positive influence parameters significant at the 0.05 level. Nodes are labeled according to their treaty numbers and colored according to topic. The S1 Appendix contains a key linking treaty ID numbers to their corresponding names. The size of the edges is proportional to the relative degree of influence the sender exercises over the receiver. One of the largest relationships is between the Convention on Biological Diversity (treaty # 40794) and the Convention on Wetlands of International Importance (treaty # 40602), indicating that the a state’s ratification of the former makes their ratification of the latter more likely.

Treaties are often linked to one another. The objectives and mechanisms of two agreements may be synergetic, complementary, or closely related, in which case the benefits (or costs) associated with ratifying one treaty may be magnified (or mitigated) if a linked treaty has previously been ratified. In addition, the ratification of one treaty may lead to the ratification of another through issue linkage, or the creation of a subsequent protocol. In other words, as states positively influence one another, so may treaties. The positive treaty influence network reveals cases of treaty linkage, wherein a state’s ratification of a given treaty means that they are more likely to ratify a different treaty. The structure for the positive treaty influence network (Fig 4), inferred using the BLIN model and then assessed through an ERGM, generally mirrors that of the negative treaty influence network. The network is rather sparse, with few treaties influencing others. As in the case of the negative influence treaty network, we obtain negative estimates for the edges and GW-degree terms. This indicates that treaties are likely to be influenced by or influence relatively few others.

### Spillover effects and substantive interpretation

To illustrate the power of state and treaty influence in global environmental politics, we examine the changes in the BLIN predictions when switching one of the 30,132 state-treaty pairs from “on” to “off.” Given its historical importance, we compare the results from a reality-based model where the US ratifies the United Nations Framework Convention on Climate Change (UNFCCC, treaty #40477) in 1992 to a counter-factual model where the US did not ratify it. By comparing the model’s predicted probability that state *i* ratifies treaty *j* over the course of time, we can depict the influence and importance of a single ratification. Given that we are interested in the complexities of these influence networks, or how the act of ratification influences states directly and indirectly influenced by the US and treaties directly and indirectly influenced by the UNFCCC, we examine the probability that a given state ratifies a given treaty at any point between 1993 and 2000 (inclusive). This longer time period was chosen to get a full sense of how far reaching the complexities of these influence networks may be. Positive differences in the change of ratification probabilities imply that the state is more likely to ratify the treaty between 1993 and 2000 if the US ratified the UNFCCC in 1992.

**Fig 5:**
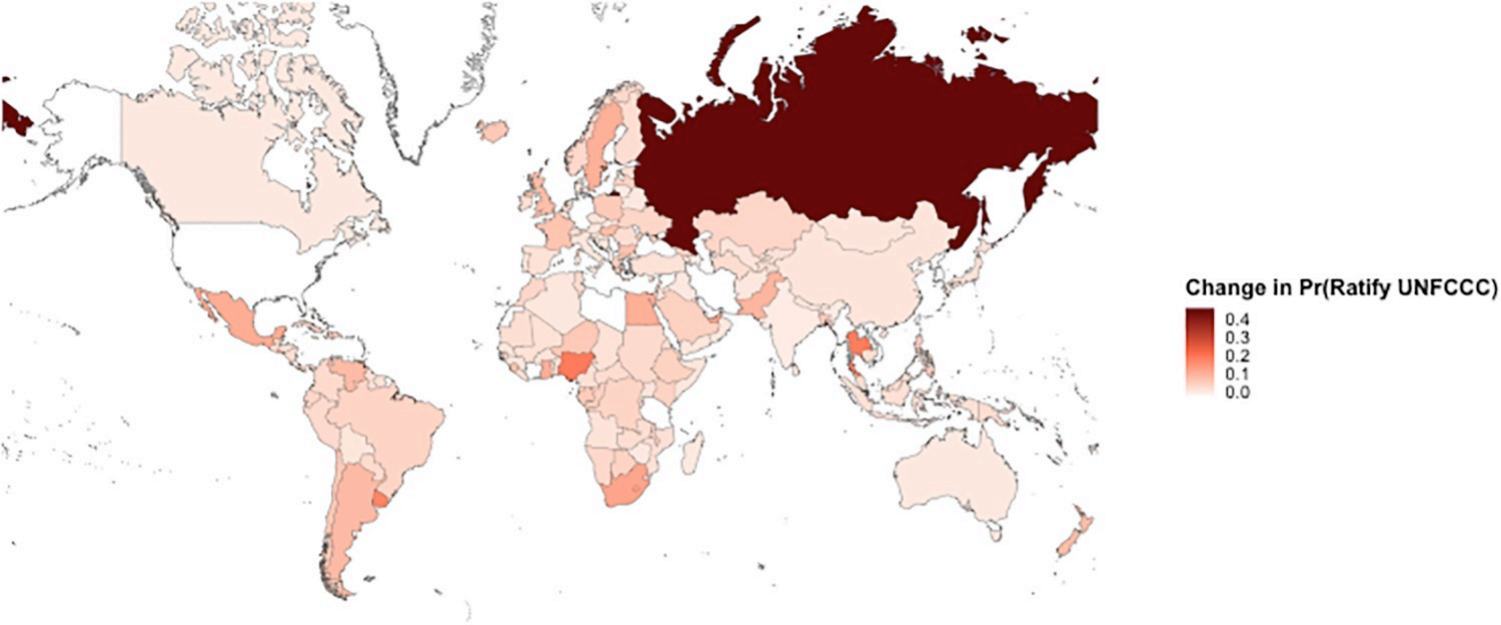
Difference in probability of UNFCCC ratification by 2000 based upon US ratification in 1992. Darker shades of red refer to larger increases in the probability of UNFCCC ratification by 2000 following the U.S.’ ratification of the UNFCC in 1992.

**Fig 6:**
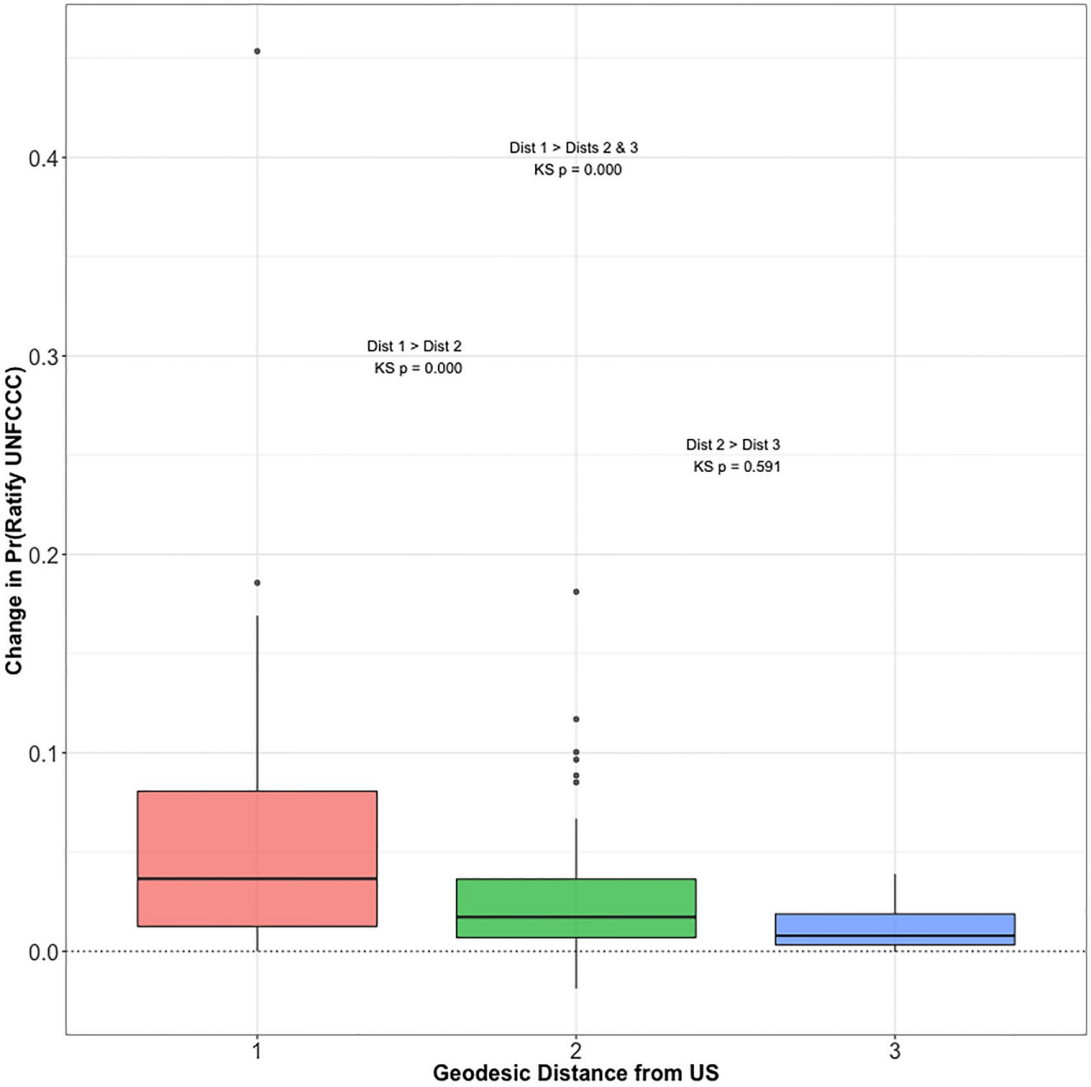
Difference in probability of UNFCCC ratification by 2000 based upon US ratification in 1992 by geodesic distance from United States. Two-sample Kolmogorov-Smirnov Tests were used to to describe the relative differences in distributions and if the distribution for direct-order alters at distance one is higher than the distributions for second and third-order alters at distances two and three respectively, or both. 35 states are directly influenced by the U.S., 143 are at distance two, and 7 are at distance three. Three tests were conducted. Two-sample KS tests for the differences between the distributions for distance one and two and two and three were conducted with KS p-values of 0.000 and 0.591 respectively. A test for the difference in distributions between distance one and distances two and three combined may be more reliable given the relatively few states at distance three. The p-value for this test is 0.000, indicating that the change in probability of ratification is significantly larger at distance one relative to distances two and three.

When examining the US’s influence through the change in the probability of UNFCCC ratification by another state (Fig 5), it appears that the direct and indirect influence exercised by the US is fairly significant. Interestingly, Russia shows the largest change in probability of UNFCCC ratification based upon the action of the US. Without US action on this treaty, Russia would have ratified the UNFCCC with a probability of around 40 percent. With US action on this treaty, though, this probability was raised to 85 percent. Interestingly, many of the states that experience changes in their probability of UNFCCC ratification, such as Russia, Nigeria, Thailand, and Uruguay, have nonexistent or insignificant influence relationships with the US. These relationships are not highlighted in Fig 2 as they are not among the significant ties. Indirect effects and the interdependencies of these influence relationships are the reason for why these states more likely to follow the US nonetheless. It would be expected that those states with which the US has a direct influence relationship experience the largest changes in the probability of ratification. However, it is possible that through longer paths, the US can still exercise indirect influence over those it would not directly affect. We find that as a state’s distance from the US increases in the positive state influence network (all edges, not just significant edges), the state’s change in probability also decreases (Fig 6). Two-sample Kolmogorov-Smirnov (KS) tests reveal that the cumulative distribution of differences in ratification probabilities for those the US directly influences is generally larger than those it exercises indirect influence over. Nevertheless, such indirect influence still exists, illustrating the complexity of international environmental governance and further underlining the importance of indirect paths, influences, and links [17]. It merits noting that the BLIN model, although it does not directly model second order dependencies (i.e., the relationship between state-treaty pairs), allows for this type of complex behavior that we expect to observe in networks [11].

**Fig 7:**
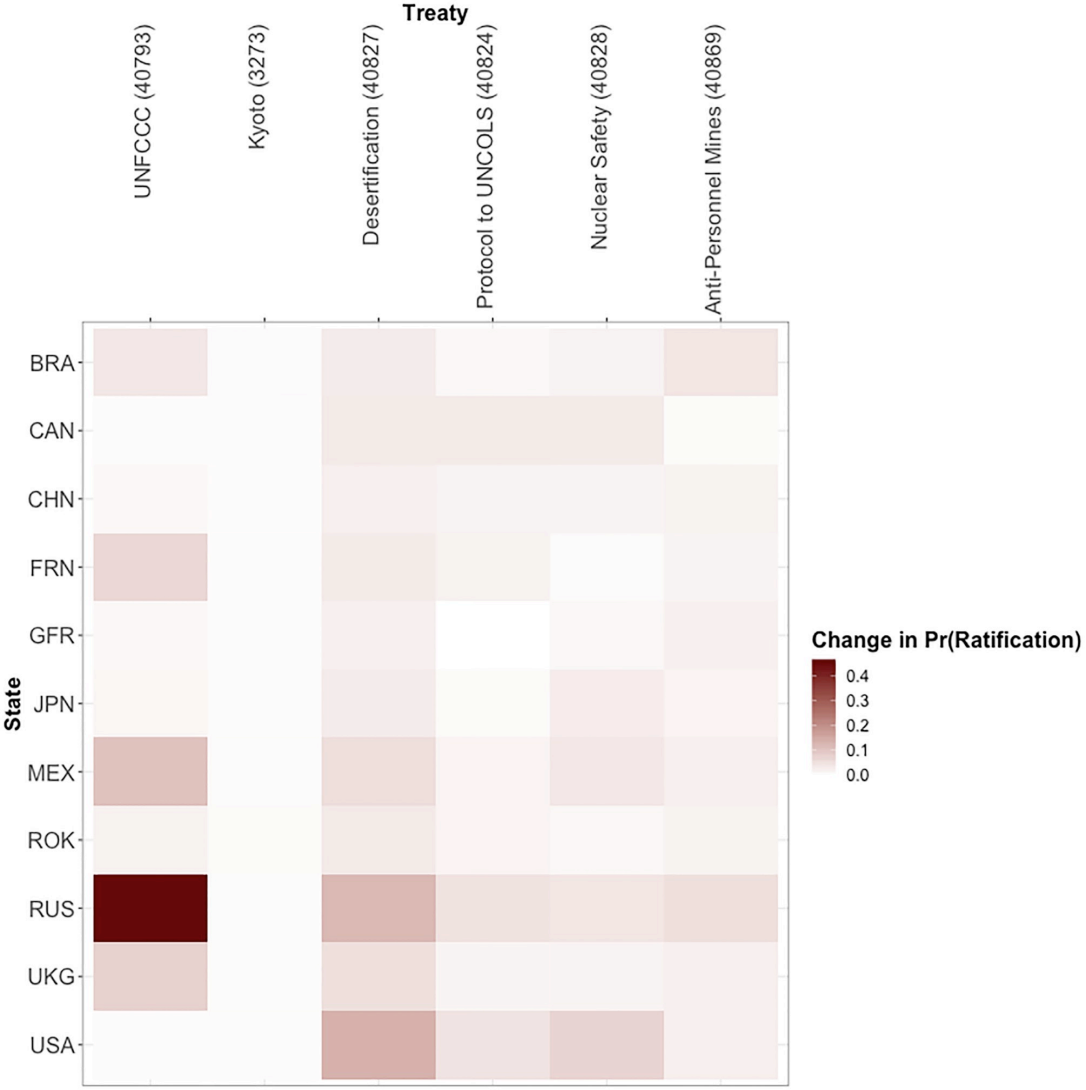
Difference in probability of different states ratifying different treaties by 2000 based upon US ratification in 1992. Darker shades of red refer to larger increases in the probability of ratification by 2000 following the U.S.’ ratification of the UNFCC in 1992. Negative values are included but are never smaller than -0.003.

Finally, to assess how the change counter-factual might spill over to different treaties, we examine the change in 11 powerful states’ probabilities of ratifying five interesting and important treaties in addition to the UNFCCC: the Kyoto Protocol (treaty # 3273), the United Nations Convention to Combat Desertification (treaty # 40827), the Convention on the Law of the Sea (treaty # 40824), the Convention on Nuclear Safety (treaty # 40828), and the Convention on the Prohibition of the Use, Production, and Transfer of Anti-Personnel Mines and on their Destruction (treaty # 40869). This analysis (Fig 7) illustrates how the simple act of a single ratification can influence powerful states to ratify treaties more, or even less, related to the agreement ratified. For example, the US’ ratification of the UNFCCC not only influences Russia’s probability of ratifying the UNFCCC, but Mexico and Russia’s probability of joining the United Nations Convention to Combat Desertification and Brazil’s probability of ratifying the Convention on the Prohibition of the Use, Production, and Transfer of Anti-Personnel Mines and on their Destruction in non-trivial ways.

## Discussion

We present and assess latent influence networks underlying international politics. The implications of this study matter for a variety of reasons. Most importantly, our findings constitute a systematic demonstration of an underlying latent influence network in international politics, including its geometry, structural features, and interdependencies. This complex network of influencer-influenced relations has long been considered, but now may be used to study a variety of international and domestic outcomes. The networks presented provide means of answering numerous foundational questions in the social sciences, including whether material resources imply influence, or whether power is truly the currency of international politics.

By examining the structure of this particular network, we shed light on the collective action dynamics—including cooperation and negative influence—surrounding one of the most important issues of our time: environmental protection. This provides practitioners the knowledge necessary to enhance their ability to mitigate environmental threats, including climate change, by promoting the ratification of essential environmental accords. State influence is likely to vary by scope and the influence network in other areas such as trade or human rights may be subject to somewhat different dynamics. Yet, the network presented here represents an essential first-cut. We have demonstrated that the BLIN model increases our ability to understand the factors influencing treaty ratification by the dramatic improvements in both model fit and predictive performance.

Having better knowledge of the role of power and influence within international politics and environmental treaty ratification is of the utmost importance. Resolving or mitigating transnational environmental problems requires cooperation in the face of collective action problems. Through understanding the patterns of influence relationships that inform whether states ratify environmental treaties, we may improve the chances for cooperation. The prioritization of individual gains to collective costs is not sustainable, and the reversal of this dilemma is only possible through understanding the interdependencies in state action.

## Supporting information

S1 Appendix Supplementary materials appendix. This document contains additional information associated with the manuscript “Latent influence networks in global environmental politics.” This includes information on the dataset and variables used, comparison to a control-only baseline logistic regression model, comparison to the baseline model of Spilker and Koubi (2016), a brief overview of the BLIN model, summary statistics for the inferred influence networks, ERGM goodness of fit diagnostics, and a discussion of European policy coordination.
(PDF)
