# Supplementary material for: Latent influence networks in global environmental politics
Source: PLoS One. 2019 Mar 7;14(3):e0213284. doi: 10.1371/journal.pone.0213284 (PMC6405053; doi:10.1371/journal.pone.0213284)
Supplement: S1 Appendix — This document contains additional information associated with the manuscript “Latent influence networks in global environmental politics.” This includes information on the dataset and variables used, comparison to a control-only baseline logistic regression model, comparison to the baseline model of Spilker and Koubi (2016), a brief overview of the BLIN model, summary statistics for the inferred influence networks, ERGM goodness of fit diagnostics, and a discussion of European policy coordination. (PDF) [file pone.0213284.s001.pdf]

Latent influence networks in global environmental politics: Supplementary Materials

Benjamin W. Campbell,[1*] Frank W. Marrs,[2] Tobias Böhmelt,[3]
Bailey K. Fosdick,[2] Skyler J. Cranmer[1]
[1]Department of Political Science, The Ohio State University
[2] Department of Statistics, Colorado State University
[3] Department of Government, University of Essex
*To whom correspondence should be addressed; E-mail: campbell.1721@osu.edu.

Description of the Dataset

Our model specification, and most of our covariate data, is sourced from Spilker and Koubi (2016) (*1*) who build upon data collected by Bernauer et al. (2013) (*2*). The units of observation are country-treaty-year triplets measured from 1972 to 2000, and an observation is included if a state has the potential to ratify a particular treaty in the year of observation. A country-treaty unit is included in the dataset until it has ratified the treaty. In this section, we will briefly review these variables, from where they are initially sourced, and their distributions.

The dataset of interest is constituted by $658,631$ country-treaty-years measured on an annual basis between 1972 and 2000. For a county-treaty-year to be included, the country has to have had the potential to ratify the treaty in that given year. The dependent variable for the baseline logit is an indicator for whether a state ratified the treaty in the year of observation. Since 1972, the number of ratifications occurring per year has increased dramatically. This dependent

variable appears to be constituted by a rare events data generating process (DGP), with only 5,927 instances of ratification. This is to say, in the potential years a state could ratify a treaty, a ratification only occurs 0.8% of the time

Any observations with missing values were case-wise deleted. Most of these observations were micro-states that are typically excluded by most analyses. In this case, multiple-imputation could not be trusted as there was relatively little information to train an imputation model on.

The Polity variable is a reference to how democratic a state is according to the "Polity" scale. This particular item is the Polity IV item and is sourced from the Polity IV Project (*3*). Polity ranges from -10 to 10. However, some values have not been converted, and as such, deserve additional attention here.

- -66: Cases of foreign interruption. It is not clear how these should be treated. Typically they're treated as "system missing".
- -77: Cases of anarchy. Typically these are treated as 0.

These special values, -66 and -77, were recoded to zero values. Beyond the above problematic values that merit recoding, there are $65,850$ instances of missingness. We include this variable as per standard practice (*2*).

The Hard Law Treaty variable is an indicator for whether or not a treaty is an instance of legally binding legal instruments. Typically these treaties exist in contrast to "soft law" treaties, which typically lack enforcement mechanisms. In this variable, there is no missingness. This data is sourced from Bernauer et al. (*2*). There are $278,873$ observations that include a hard-law treaty (values of 1), and the remaining $379,758$ observations which include soft-law treaties (values of 0).

The Legislative Approval variable is an indicator for whether a treaty has support in the legislature. This variable is sourced from Hathaway (*4*). In this variable, the breakdown of

values is as follows:

- 0: $173,277$
- 1: $465,988$
- NA: $19,366$

Logged SO_2 PC is a variable that captures the amount of SO_2 a country produces and is typically thought to be a reference to the country-based production of industrial pollution. The summary of this variable's distribution is as follows:

- Min: -19.17
- Median: -12.12
- Mean: -12.03
- Max: -6.04
- NAs: $100,865$

Logged Real GDP PC is a reference to the extent of economic development that a state possesses for a given year. The distribution of this variable is as follows:

- Min: 5.64
- Median: 8.20
- Mean: 8.22
- Max: 10.74
- NAs: $55,862$

We include this variable as per standard practice (*5*).

Assistance is a treaty-level variable that indicates whether a given treaty provides assistance in an effort to comply with the treaty. This variable is constituted by $54,822$ instances of treaty-based assistance (or 1s), and $603,809$ observations without assistance (or 0s). There are no cases of missingness. Assistance to Developing Countries is a treaty-level variable that indicates whether a given treaty provides assistance to developing countries in an effort to comply with the treaty. There are $28,748$ observations where treaties mandate assistance to developing countries and $629,883$ cases where they do not. There is no missingness in this variable.

Logged Openness is a country-level variable that references the degree of trade openness for a country. We include this variable as suggested (*2*). It is calculated as follows:

$$Openness = log(\frac{Imports + Exports}{GDP}) \tag{1}$$

This variable is calculated using the Gleditsch (*6*) GDP and trade data, which is expanded to include data for all independent states. The distribution of this variable is as follows:

- Min: -21.14
- Median: -15.66
- Mean: -15.36
- Max: 0.89
- NAs: $55,862$

IGO membership is a variable taken from the Correlates of War (*7*) dataset on IGOs. This variable counts the number of IGO memberships that each county possesses for each year. This variable is standard in many models (*2*). The distribution of this variable is as follows:

- Min: 0

- Median: 48.00
- Mean: 48.89
- Max: 129.00
- NAs: $26,339$

The Number of Countries Ratified variables is calculated as the lagged number of countries that have previously ratified the treaty in question. The distribution of this variable is as follows:

- Min: 0
- Median: 13.00
- Mean: 19.9
- Max: 178.00

The Percent of Region Ratified is calculated as the lagged percent of countries in the same region that have ratified the treaty in question up to the following year. This variable is distributed as follows:

- Min: 0
- Median: 0
- Mean: 7.327
- Max: 94.120

The Percent of Income Group Ratified is calculated as the lagged percent of countries in the shared income group that have previously ratified the treaty in question. The distribution of this variable is as follows:

- Min: 0
- Median: 2.899
- Mean: 7.956
- Max: 97.140

Logged GDP is calculated as the natural log of a country's GDP for a given year, and accounts for the role of economic development on whether a state ratifies a treaty. This data is sourced from Gleditsch (*6*). The distribution of this variable is as follows:

- Min: 19.44
- Median: 23.67
- Mean: 23.95
- Max: 29.85
- NAs: $67,788$

Following the recommendations of Carter and Signorino (2010) (*8*), we specify three functions of time to account for temporal dependence. These three functions include the number of years that a given state has had to ratify, then that number squared and cubed. For the t^1 variable, the distribution is as follows:

- Min: 0
- Median: 12.0
- Mean: 14.62
- Max: 50.0

For the t^2 and t^3 variable distributions, one must only take the relevant exponential function of the variable.

Variable Collinearity

Figure 1 presents a visualization and discussion of the correlation between variables included in the manuscript model. We examined possible collinearity by calculating the Variance Inflation Factor (VIF) for all variables; a high VIF indicates that a variable's standard error is inflated due to collinearity. Only five variables have a (VIF) that is greater than four, three of which are the temporal dependency variables (which is to be expected as they are simple exponential functions of one another). The remaining two variables with VIF above four are the number of countries ratifying the treaty and the number of countries in the same income group ratifying a treaty. The VIFs of these variables are 8.709220 and 8.022883, respectively. The high correlation indicated between these variables is not particularly concerning as one is not interested in substantively interpreting the standard errors associated with them.

Baseline Logistic Regression Model

Table 1 presents the results from a baseline logistic regression model including the variables of interest. This specification mirrors that of Spilker and Koubi (*1*) but with an updated dataset. It also mirrors the penalized logistic regression model presented in the manuscript but does not include penalization and does include robust standard errors clustered by country. Overall, results fall in line with what may be expected:

- Countries are less likely to ratify treaties that require constraints and are examples of "hard law."
- Interestingly, countries with a relatively large amount of sulfur dioxide pollution are more

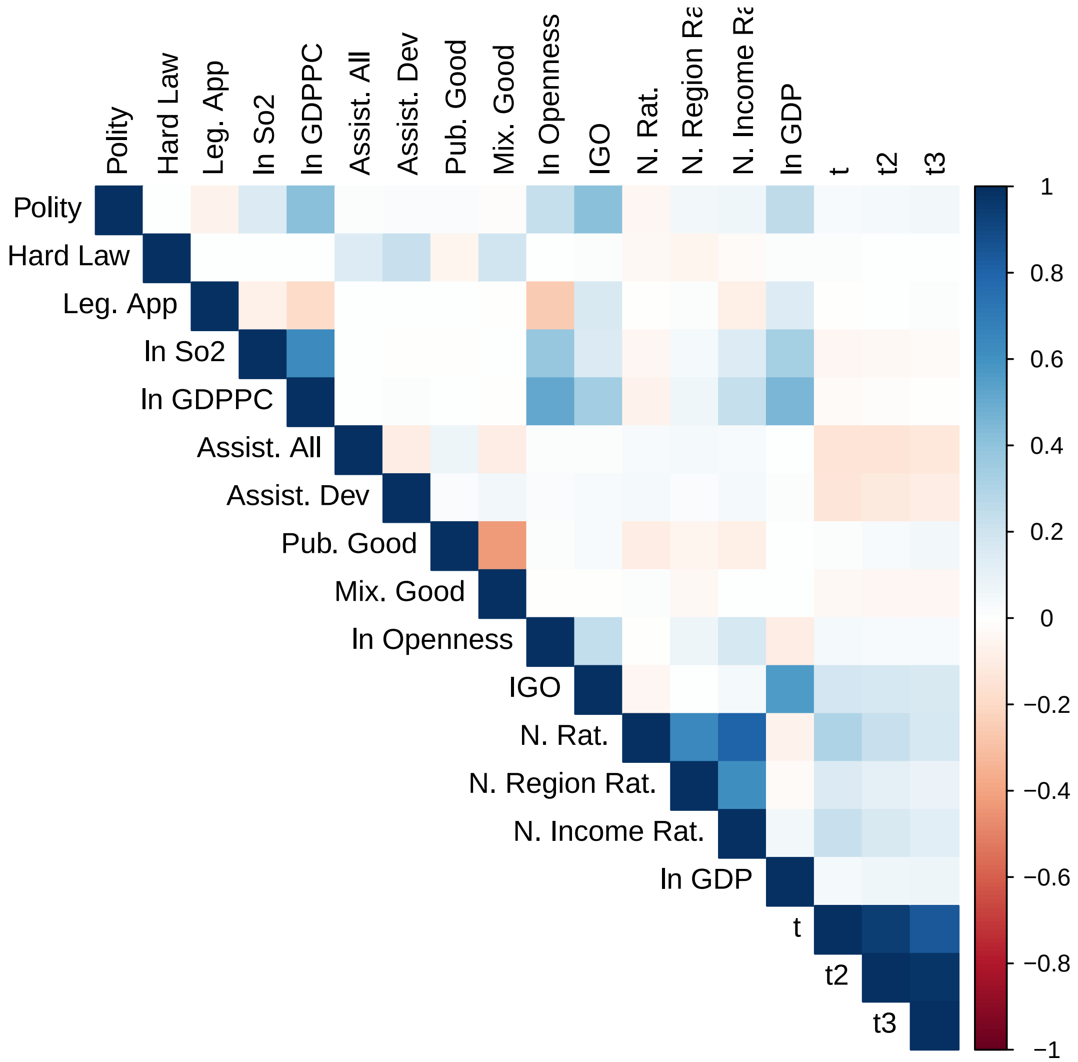

Figure 1: **Correlation Matrix.** Correlation heat-map with cells coloured according to the correlation between explanatory variables; these variables are described in Materials and Methods. This figure leads us to believe that overall, the correlation between control variables does not produce perfect collinearity or any degree of collinearity capable of biasing estimates. While there is some unavoidable correlation between the economic terms (e.g. openness, GDP, GDP PC) or the temporal baselines, each variable is necessary as each reflect a different theoretical process.

likely to ratify a treaty, as are those with a large degree of economic development, as per the GDP PC term.

- Treaties that provide assistance to all countries or to developing countries are both more likely to be ratified.
- Treaties pertaining to global public and mixed goods are less likely to be ratified.
- While countries that are economically open are less likely to ratify environmental treaties, countries that are politically open (as per IO memberships) are more likely to ratify treaties.
- Peer effects based upon all countries that have ratified a treaty and those in the same region and income group positively influence treaty ratification.

Comparison to Spilker and Koubi (2016)

Results for the baseline model are presented in Table 1. This model specification resembles the baseline specification of Spilker and Koubi (2016) Model 1. Variables colored in blue reflect the same sign and significance as Spilker and Koubi, while variables colored in red represent departures. The following variables share direction and significance level with the Spilker and Koubi findings:

- Intercept (negative and significant)
- Hard Law Treaty (negative and significant)
- SO_2 PC (positive and significant)
- Assistance (positive and significant)

	Baseline Model
(Intercept)	-6.62*
	(0.91)
Polity	0.01
	(0.01)
Hard Law	-0.16*
	(0.04)
Legislative Approval	-0.04
	(0.09)
ln SO_2 PC	0.11*
	(0.03)
ln GDP PC	0.08
	(0.05)
Assistance (All)	0.46*
	(0.05)
Assistance (Developing Countries)	0.78*
	(0.07)
Global Public Goods	-0.45*
	(0.04)
Global Mixed Goods	-0.16*
	(0.06)
ln Openness	-0.10*
	(0.05)
IO Membership	0.02*
	(0.00)
Countries Ratified	0.01*
	(0.00)
Countries in Region Ratified	0.03*
	(0.00)
Countries in Income Group Ratified	0.02*
	(0.00)
ln GDP	0.05
	(0.03)
t	-0.32*
	(0.02)
t^2	0.01*
	(0.00)
t^3	-0.00*
	(0.00)

Table 1: **Baseline Logit of Controls.** No penalization with robust errors clustered by country. Starred coefficients are significant at the $p < 0.05$ level. Variables colored in blue reflect the same sign and signficance as Spilker and Koubi, while variables colored in red represent variables with different sign or significance.

- Assistance Developing Countries (positive and significant)
- Global Public Good (negative and significant)
- Global Mixed Public Good (negative and significant)
- N of Countries Ratified (positive and significant)
- N of Countries in Same Region (positive and significant)
- t (negative and significant)
- t^2 (positive and significant)
- t^3 (negative and significant)

The following variables are not in the expected direction with the expected significance level:

- Polity (null effect, expect positive and significant)
- Logged GDPPC (null effect, expect positive and significant)
- Legislative Approval (negative and non-significant, expect positive and non-significant)
- Logged Openness (negative and significant, expect positive and non-significant)
- N of Countries in Same Income Group (positive and significant, expect negative and non-significant)
- Logged GDP (null, expect positive and significant)
- IO membership (positive and significant, expect negative and non-significant)

These discrepancies are unclear. The variables that switch signs are those that typically already had low effect sizes. However, it's entirely possible we are uncovering idiosyncrasies in the data of Spilker and Koubi that are changing when we go to a more reliable sample.

Overview of the BLIN Model

In this section we detail the fitting of the BLIN model. A key property of this model is that it may be written as a linear regression,

$$
\begin{aligned}
g\big(E[\mathbf{y}_t|\mathbf{Y}_{t-}]\big) &= ((\sum_{k=1}^{3} \mathbf{Y}_{t-k})^T \otimes \mathbf{I}_S)\text{vec}(\mathbf{A}^T) \\
&\quad + (\mathbf{I}_L \otimes \sum_{k=1}^{3} \mathbf{Y}_{t-k})\text{vec}(\mathbf{B}) + \mathbf{Z}_t\beta, \\
&:= \mu + \mathbf{X}_B^t \boldsymbol{\theta},
\end{aligned} \tag{2}
$$

where $g(x) = \log\big(\frac{x}{1-x}\big)$ is the logit function applied element-wise, $\mathbf{y}_t$ is the columnwise vectorization of response $\mathbf{Y}_t$ at time t, $\mathbf{I}_n$ is the $n \times n$ identity matrix, $\mathbf{Z}_t$ is a matrix of covariates, and $\mathbf{X}_B^t$ is the composite design matrix with coefficients $\boldsymbol{\theta} = [\text{vec}(\mathbf{A}), \text{vec}(\mathbf{B}), \beta]^T$.

As social networks are generally sparse, one would expect that many influence relationships are relatively small or non-existent. Likewise, we expect small or zero values for many entries in the $\mathbf{A}$ and $\mathbf{B}$ matrices. For example, the signature of treaties by Zambia may have no influence of the signature of treaties by the Netherlands. As our model is significantly more flexible than the covariate-only baseline, we must guard against the possibility of overfitting. Thus, to induce parsimony and guard against overfitting, we augment the usual maximum likelihood approach with a sparsity-inducing penalty. Assuming each $y_{ij}^t|\mathbf{Y}^{t-1}, \mathbf{Z}_t$ is an independent Bernoulli random variable with expectation given in (2), the penalty increases the negative log-likelihood the farther $\boldsymbol{\theta}$ is away from zero. We select the Lasso penalty of Tibshirani (1996) (*9*), which uses

the L^1 norm to assess the distance of $\boldsymbol{\theta}$ from zero. This penalty forces some entries in $\boldsymbol{\theta}$ to zero, effectively performing variable selection and regularization simultaneously. Our objective function for the sparse BLIN model becomes

$$\{\widehat{\boldsymbol{\theta}}, \widehat{\mu}\} = \underset{\mu, \boldsymbol{\theta}}{\text{argmin}} \sum_t h(\mathbf{y}_t, \mathbf{X}_B^t, \mu, \boldsymbol{\theta}) + \lambda ||\boldsymbol{\theta}||_1, \tag{3}$$

$$\text{for } h(\mathbf{y}_t, \mathbf{X}_B^t, \mu, \boldsymbol{\theta}) = \sum_{i,j} \log(1 + \exp(\mu + \boldsymbol{\theta}^T \mathbf{x}_{ij}^t)) - y_{ij}^t(\mu + \boldsymbol{\theta}^T \mathbf{x}_{ij}^t) \tag{4}$$

where λ is a tuning parameter to be selected, h is the negative log-likelihood for independent Bernoulli random variables, and $\mathbf{x}_{ij}^t$ is the row in $\mathbf{X}_B^t$ corresponding to state i and treaty j. Larger values of λ force more entries in $\boldsymbol{\theta}$ to zero. Notice that there is no penalty on the global mean μ in this procedure.

We solve the optimization problem in (3) using the `glmnet` package in `R`. This package computes a family of solutions for a grid of values of λ using coordinate descent; for more details on this solution procedure, see Friedman et al. (*10*). To select λ we perform 10-fold cross-validation using the area under the precision-recall curve (AUCPR) as the measure of model fit. As this measure is not implemented in `glmnet`'s native cross-validation function, we perform our own cross-validation as is described in Algorithm 1. We select the λ value that gives maximum mean out-of-sample AUCPR, where the mean is over all 10-fold cross-validations.

In addition to writing our own cross-validation, we find that the estimation of the model in (3) using `glmnet` fails to converge for some partitions of the data. We postulate that this is a result of the ratifications of environmental treaties being sparse; approximately 1% of possible country-treaty-year ratifications are actually ratified. We pruned the data such that the fitting of the model in (3) using `glmnet` converges. To start, we removed all countries that ratify fewer

than 4 treaties and all treaties that are ratified by fewer than 4 countries, resulting in the removal of 3 countries and 36 treaties from the data set. We postulate that there is too little information in the data to make inferences regarding these countries and treaties. In addition, we suggest that there may be country pairs and treaty pairs for which there is too little information to make inference. For example, to infer the relationship between the USA and Germany, Germany must have the opportunity to sign some treaty in the three years following the ratification of said treaty by the USA. When the number of the opportunities is few, we may have too little information to infer the influence of the USA on Germany. In addition to removing countries (and treaties) that ratify (and are ratified) fewer than 4 times, we also removed entries in $\boldsymbol{\theta}$ for which there are fewer than $n_i = 4$ opportunities for inference. We repeat the fitting algorithm, increasing n_i by 1 until the fit of the full dataset converges for at least 25 values of λ and each cross validation converges for at least 90% of the full fit λ values. For details, see Algorithm 1. For the fits presented in this paper, we have removed all influence pairs for which there are fewer than or equal to 6 opportunities for inference.

Once we have coefficient estimates, it is desirable to evaluate the significance of the estimates. Selection for inclusion in the model of a given coefficient is not necessarily evidence of its significance. We use the `selectiveInference` package in `R` to generate asymptotically-valid p-values for each nonzero coefficient, where the asymptotic regime is T tending to infinity with S and L fixed. We recognize coefficients as significant when $p < 0.05$. For more details on the calculation of post-selection p-values, please see Taylor and Tibshirani (*11*) and Lee et al. (*12*). We made slight modifications to the `selectiveInference` package to allow for the use of sparse design matrices $\mathbf{X}_B^t$.

Algorithm 1 Fitting procedure for BLIN model.

$n_i \leftarrow 4$
$indicator \leftarrow$ `FALSE`
while $indicator =$ `FALSE` **do**
    Remove coefficients from model for which there are fewer than n_i opportunities for inference
    Fit full model with `glmnet`
    Save list of λ values for which fit converged in `glmnet` into list l_λ
    if `length`$(l_\lambda) > 25$ **then**
        Partition data randomly into 10 partitions, $\mathcal{D}_j$ for $j \in \{1, 2, \ldots, 10\}$
        Initialize $10 \times$ `length`(l_λ) array A
        for $j \in \{1, 2, \ldots, 10\}$ **do**
            Fit model to $\mathcal{D}_{-j}$, the training data (i.e. that without partition $\mathcal{D}_j$) at values in l_λ
            Measure performance of fitted coefficients on test set $\mathcal{D}_j$ using AUCPR
            Store AUCPR for all converged λ values in the ith row of A
        end for
        if Fit converged for 90% or more entries in l_λ for ALL partitions $\mathcal{D}_j$ **then**
            $indicator \leftarrow$ `TRUE`
        end if
    end if
end while

Predictive Performance for the BLIN Model

In fitting the entire environmental treaty ratification dataset, we found that the BLIN model explained significantly more variation (as measured by within-sample AUCPR) than the baseline logistic regression model. However, this measure does not suggest that the BLIN model is representative of the true data generating process. In other words, the BLIN model's network coefficients (the entries in the $\mathbf{A}$ and $\mathbf{B}$ matrices) may simply model residual variation in the data set beyond the baseline covariates rather than systematic network effects. To evaluate the efficacy of the BLIN model for the environmental treaties dataset, we fit the BLIN model using the algorithm above to all 10 year and 20 year subsets of the data. We then measured the in-sample performance (AUCPR over the subset) and out-of-sample performance (AUCPR for the fitted model on the following year) of the BLIN model. For example, we fit the BLIN model to the data from years 1972 to 1981, computing AUCPR on these years (in-sample). We then estimate the likelihood of each ratification in 1982 and compute AUCPR for this estimation (out-of-sample). We repeat the process for 1973 to 1982, and so on up to 1990 to 1999. We compare the performance of the BLIN model to the baseline logistic regression model in Figure 2. We see that the BLIN model explains much more of the variation of the data within sample while predicting as well out-of-sample. This suggests that the BLIN model, rather than overfitting, is explaining systematic network effects of environmental treaty ratification.

Counter-Factual study

We compared the results from a reality-based model where the US ratifies the United Nations Framework Convention on Climate Change (UNFCCC, treaty #40477) in 1992 to a counterfactual model where the US did not ratify it. To determine the probability that a given country ratified a given treaty between 1992 and 2000 (as in the Spillover Effects and Substantive In-

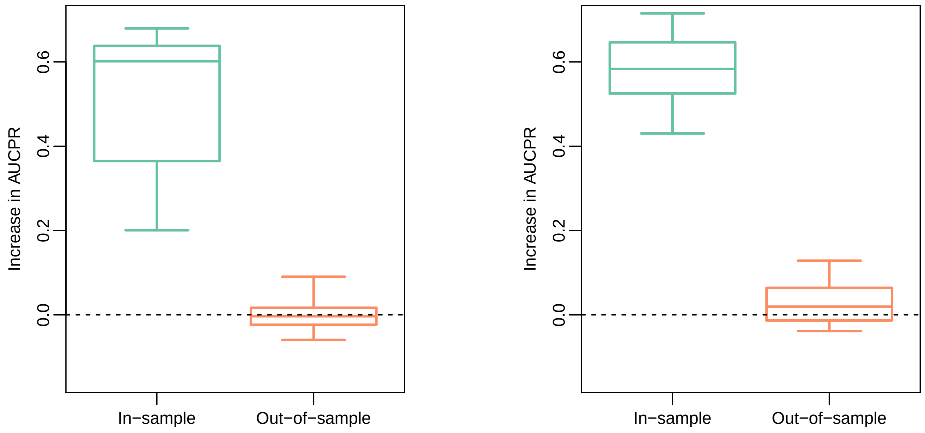

(a) 10 year subsets (b) 20 year subsets

Figure 2: **Predictive Performance.** Increase in AUCPR when fitting the BLIN model compared to the baseline logistic regression. We see that the BLIN model explains much more of the variation of the data within sample while predicting as well out-of-sample. This suggests that the BLIN model, rather than overfitting, is explaining systematic network effects of environmental treaty ratification.

terpretation section), we ran 10,000 Monte Carlo simulations for each ratification statues of interest; that is, when the US entered UNFCCC in 1992 and when the US did not. We used the point estimates of $\mathbf{A}$, $\mathbf{B}$, and β to estimate the probability that all available country-treaty pairs were ratified in 1993, and then drew a random instance of ratifications from these probabilities. We then used the instance of ratifications in 1993 to repeat the procedure for 1994, and so on until 2000. This procedure constituted one simulation. We estimated the probability that a given country-treaty pair ratified between 1993 and 2000 as simply the number of times the pair was ratified divided by the total number of simulations. The procedure used to perform the Monte Carlo simulations is given in Algorithm 2. The ratification status of the UNFCCC by the US is controlled by manipulating $\mathbf{Y}^{1992}$; we note that the US is not allowed to ratify the UNFCCC under the counter-factual.

Algorithm 2 Monte Carlo procedure for counter-factual study.

Number of simulations $m \leftarrow 10,000$
Initialize estimated probabilities of ratification, $\{\widehat{p}_{ij}\} \leftarrow 0$ for $i \in \{1, 2, \ldots, S\}$ and $j \in \{1, 2, \ldots, L\}$
Initialize number of ratification for each country-treaty pair, $\{n_{ijk}\} \leftarrow 0$ for $i \in \{1, 2, \ldots, S\}$, $j \in \{1, 2, \ldots, L\}$, and $k \in \{1, 2, \ldots, m\}$
For $t < 1993$, set $\widehat{\mathbf{Y}}^t = \mathbf{Y}^t$, the observed ratifications
for $k \in \{1, 2, \ldots, m\}$ **do**
  for $t \in \{1993, 1994, \ldots, 2000\}$ **do**
    Estimate the probability that each country ratifies each treaty in the current year, $\mathbf{P}^t$, based on the previous ratifications, $\{\widehat{\mathbf{Y}}^{t-a}\}_{a=1}^{3}$
    Randomly draw an instance of ratifications for the current year, $\widehat{\mathbf{Y}}^t$, from $\mathbf{P}^t$
  end for
  Store each ratification, that is $n_{ijk} = \sum_{t=1993}^{2000} y_{ij}^t$
end for
Final ratification estimates are $\widehat{p}_{ij} = \frac{1}{m}\sum_{k=1}^{m} n_{ijk}$

Summarizing the Inferred Influence Networks

To further understand the influence networks inferred from the BLIN model presented in the manuscript, we describe the networks using many conventional measures. In particular, we focus upon five quantities:

1. Isolates: The number of countries that neither influence other countries or are influenced by other countries.

2. Network Density: This is an aggregate measure of the degree of influence relationships that exist in the network as a whole.

3. Edgecount: This quantifies the number of influence relationships that exist in the network.

4. Reciprocity: For the total number of dyads in the network, reciprocity measures the number of sent-edges that are returned by the influencer. In other words, it provides the percent of influence relationships where actors both influence and are influenced by one

	State, Negative	State, Positive	Treaty, Negative	Treaty, Positive
Isolates	104	85	191	191
Density	0.002	0.003	0.0003	0.0006
Edge Count	74	111	16	35
Reciprocity	0.0	0.0	0.0	0.0

Table 2: **Descriptive Statistics for Influence Networks.**

another.

5. Degree Distribution: We provide histograms for the distribution of in and out-degrees in the network.

Table 2 presents the descriptive statistics for each of these networks, which we discuss in the following sections.

We begin with the negative state influence network with ties subset at influence parameters significant at the 0.05 level. The descriptive statistics for this network, presented in Table 2, are summarized as follows. 149 countries exercise no significant negative influence over any other country, or have any significant negative influence exercised over them. This network has a density of 0.002, reflecting a fairly sparse network. There are 74 instances of countries exercising significant negative influence over other countries. 0% of edges are reciprocated in the negative state influence network. The degree distribution for this network is presented in Figure 3.

The positive state influence network of significant ties is described as follows (see also Table 2). 146 countries are isolates, sending and receiving no ties in the positive state influence network. This network has a density of 0.003, reflecting a fairly sparse network that is more dense than the negative state influence network. There are 111 significant influence ties in the positive state influence network, many more than in the negative state influence network. 0% of edges are reciprocated in the positive state influence network. Figure 4 presents the degree distribution for this network.

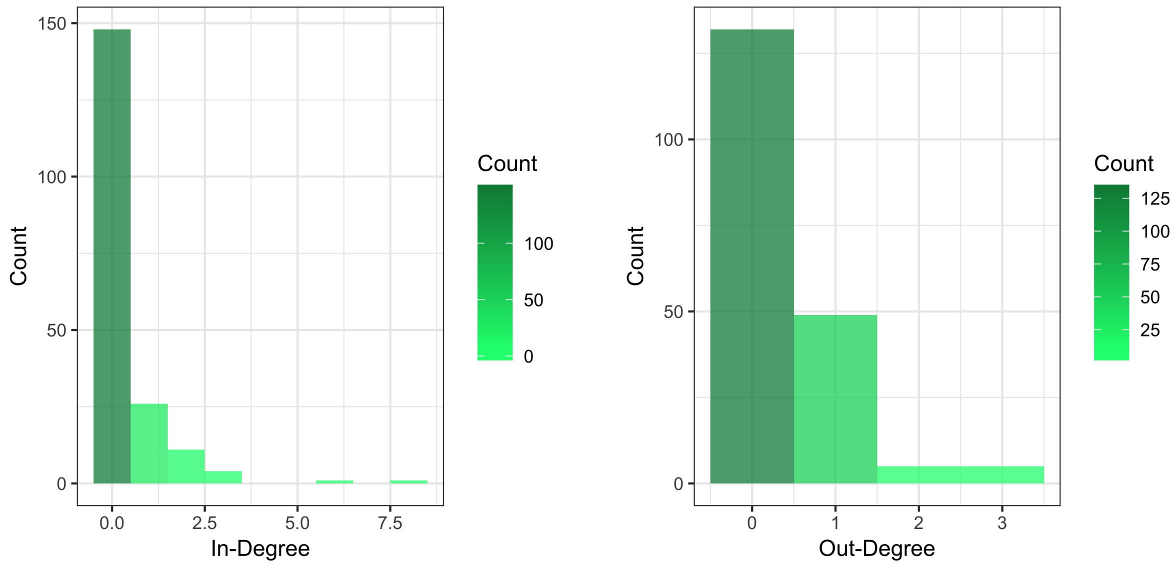

(a) In-Degree (b) Out-Degree

Figure 3: **Degree Distribution, Negative State Influence Network.** Figure indicates that influencers are only likely to exercise influence over one or two other countries, and influencees while most likely to only be influenced by one state, may at times be influenced by many.

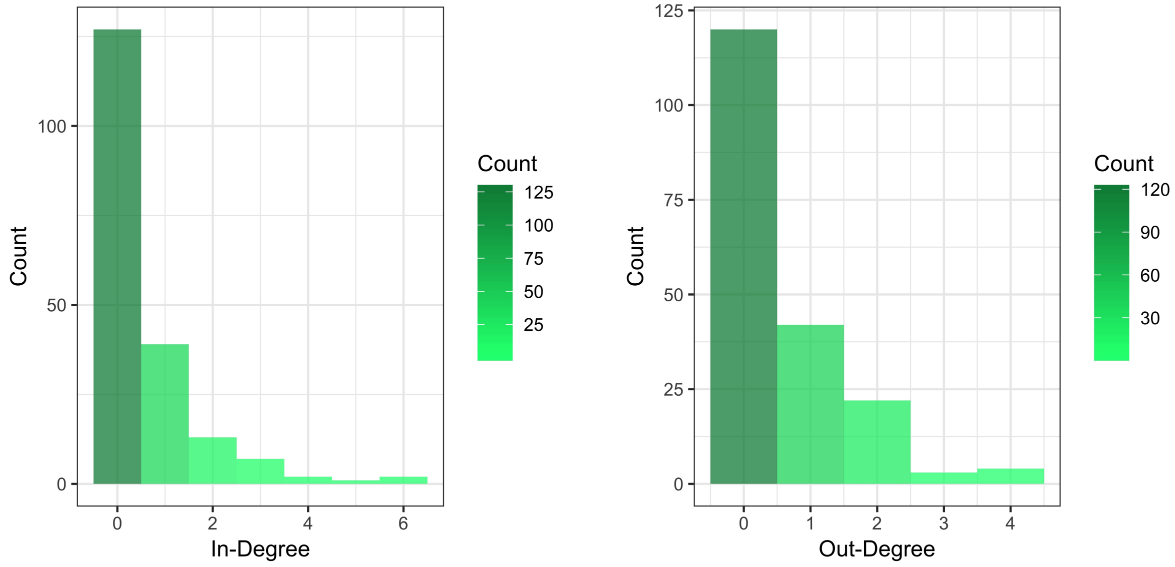

(a) In-Degree (b) Out-Degree

Figure 4: **Degree Distribution, Positive State Influence Network.** Figure demonstrates that there is much more activity in the positive state influence network relative to the negative state influence network, as the degree distribution has much more activity on the right tail.

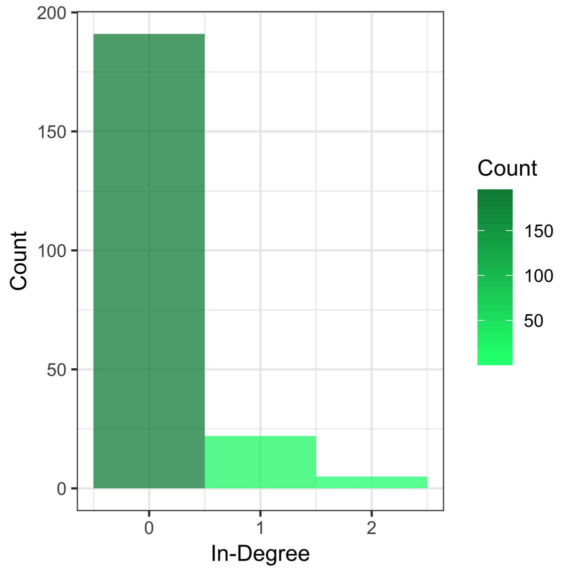

(a) In-Degree

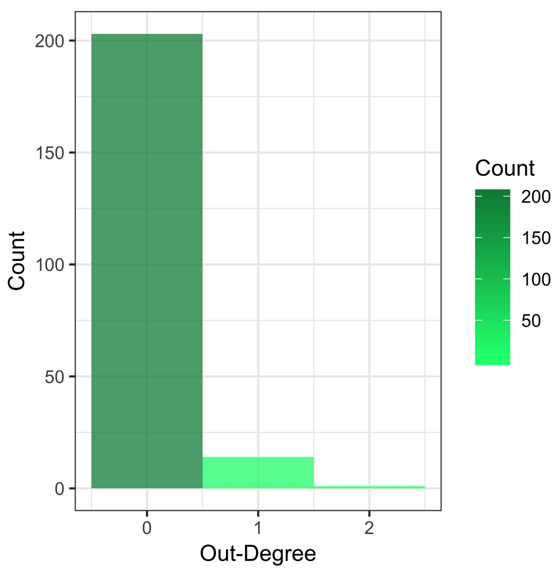

(b) Out-Degree

Figure 5: **Degree Distribution, Negative Treaty Influence Network.** Figure demonstrates that the degree distribution shows very little activity.

The negative treaty influence network, as discussed in the manuscript and described in Table 2, can be described as follows. 191 treaties are isolates, sending and receiving no ties in the negative treaty influence network. This network has a density of 0.0003, reflecting a very sparse network. There are 5 significant influence ties in the negative treaty influence network, reflecting very few instances of negative influence. 0% of edges are reciprocated in the negative treaty influence network. The degree distribution associated with the negative treaty influence network is presented in Figure 5.

The positive treaty influence network, as is presented and discussed in the manuscript and described in Table 2, is described as follows. 191 treaties are isolates in the positive treaty influence network, sending and receiving no ties. The positive treaty influence network has a density of 0.0006, reflecting a very sparse network that is still an order of magnitude more dense than the negative treaty influence network. There are 35 instances of significant influence in the positive treaty influence network. The positive state influence network has a reciprocity rate of

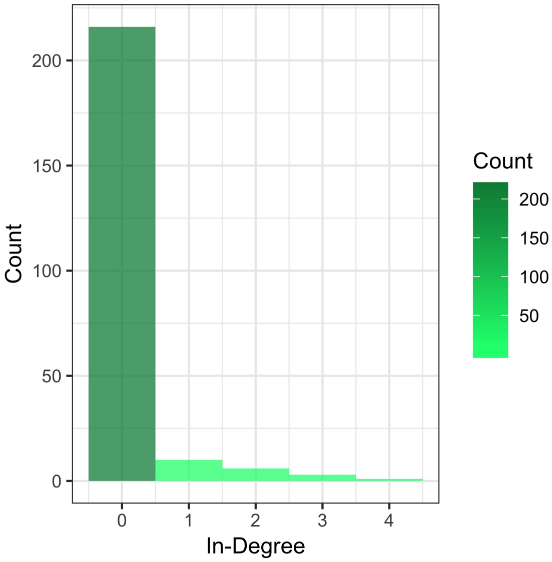

(a) In-Degree

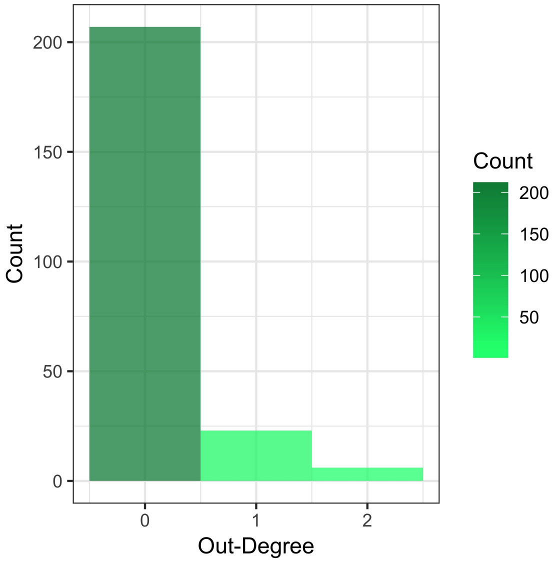

(b) Out-Degree

Figure 6: **Degree Distribution, Positive Treaty Influence Network.** This degree distribution, as expected, shows much more activity and a more spread out distribution than the negative treaty influence network degree distribution.

0%. Figure 6 presents the degree distribution for the positive treaty influence network.

Goodness of Fit for Influence Network ERGMs

To assess how well the goodness of fit for the ERGMs estimated on the influence networks, we compare networks simulated from the estimated model parameters to the observed networks (*13*). These goodness of fit diagnostics are presented in Figures 7 through 10 for the negative state, positive state, negative treaty, and positive treaty influence networks respectively. A well fitting ERGM will have observed network statistics that intersect the median statistics for the networks simulated from the estimated model. Each of these diagnostics demonstrates that the ERGMs estimated fit reasonably well.

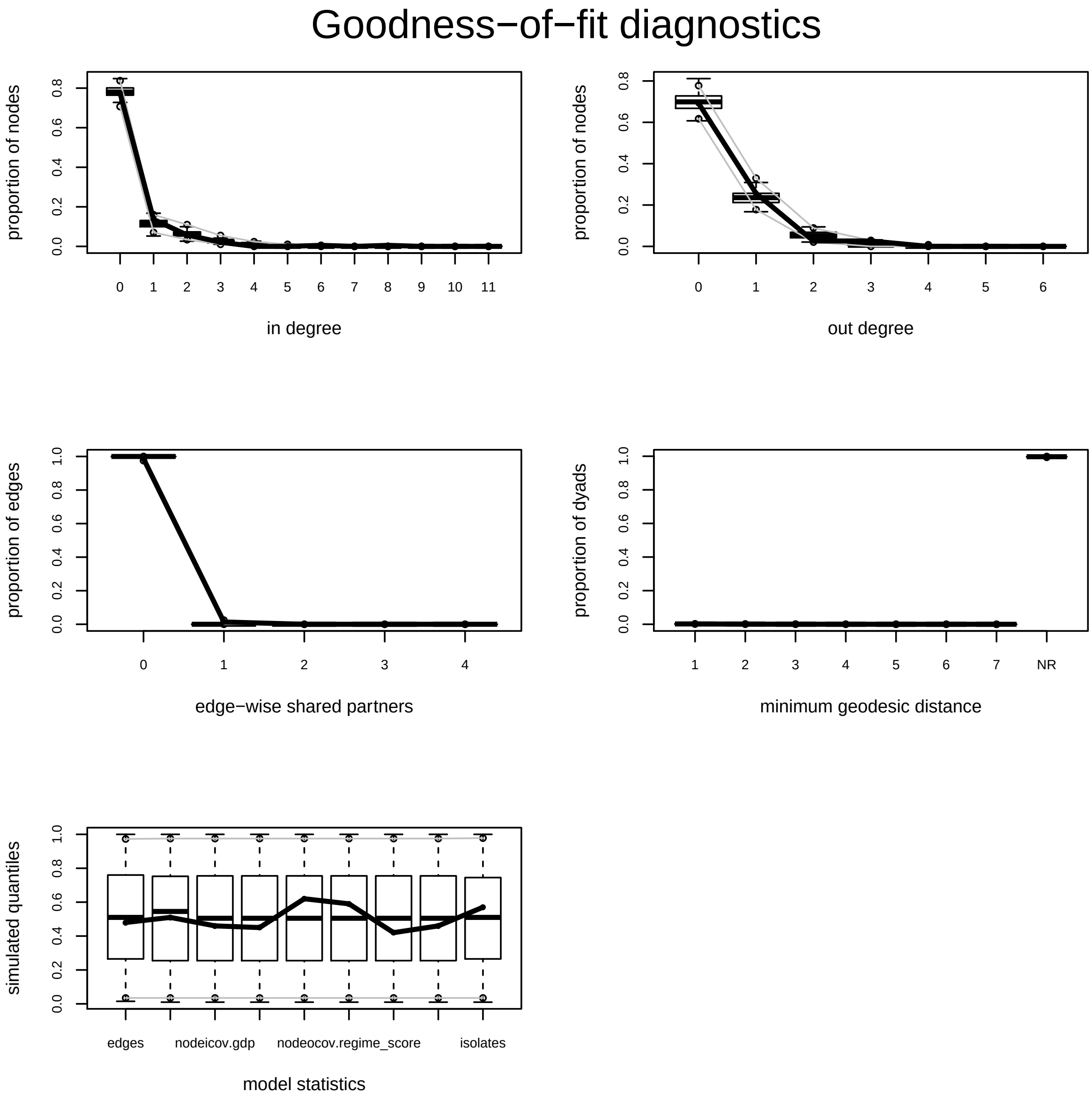

Figure 7: **Negative State Influence ERGM Goodness of Fit Boxplots.** Boxes reflect networks simulated from model estimates, line reflects the observed statistics of the network. A well fitting ERGM should have observed statistics intersecting the boxplot medians. The ERGM presented in Table 2 of the manuscript for the Negative State influence Network appears to fit very well. It fits the degree distributions of these networks particularly well.

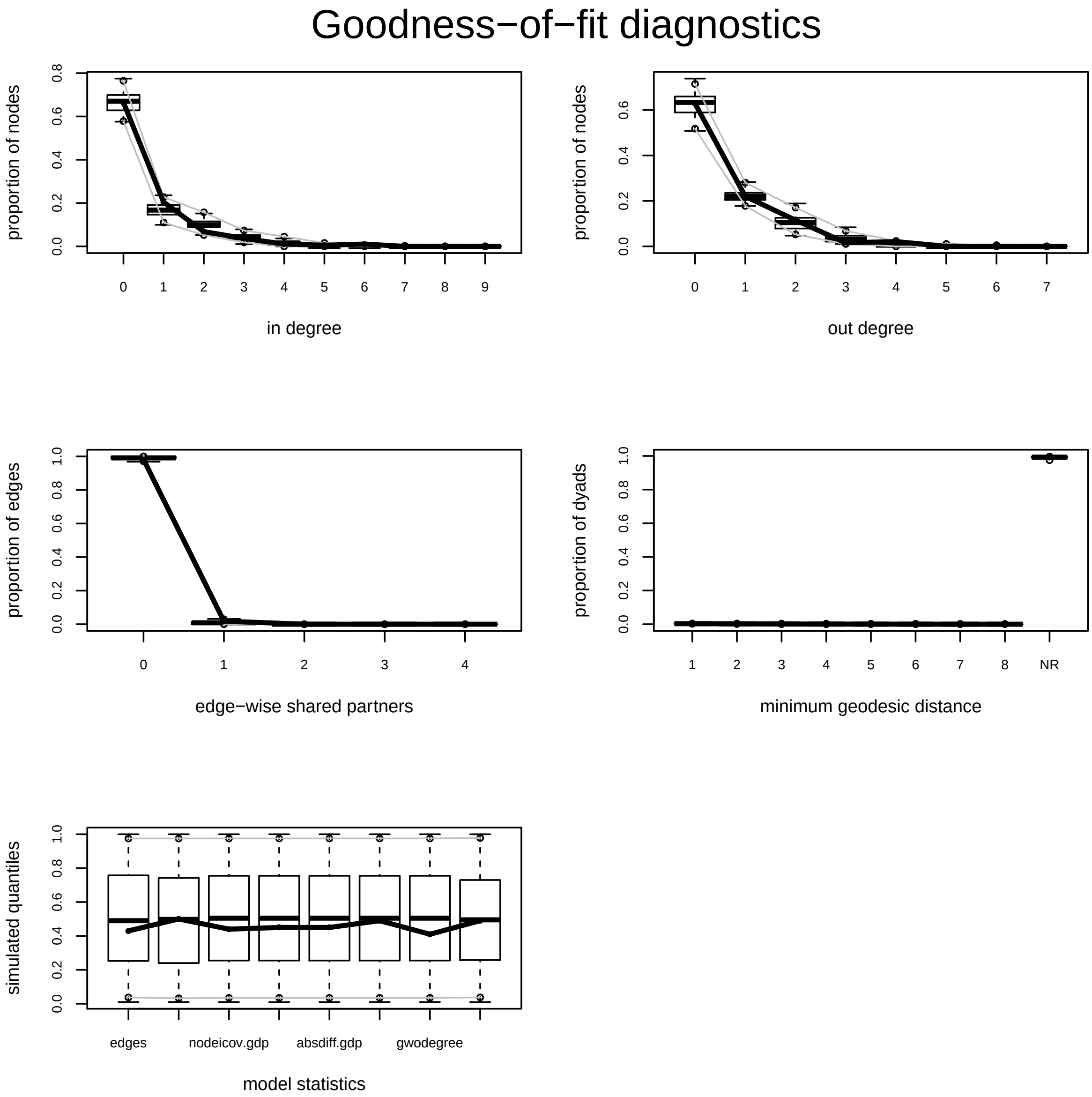

Figure 8: **Positive State Influence ERGM Goodness of Fit Boxplots.** Boxes reflect networks simulated from model estimates, line reflects the observed statistics of the network. A well fitting ERGM should have observed statistics intersecting the boxplot medians. The ERGM for the Positive State Influence Network, presented in Table 2 of the manuscript, appears to fit particularly well. This ERGM appears to fit the degree distribution of the observed network.

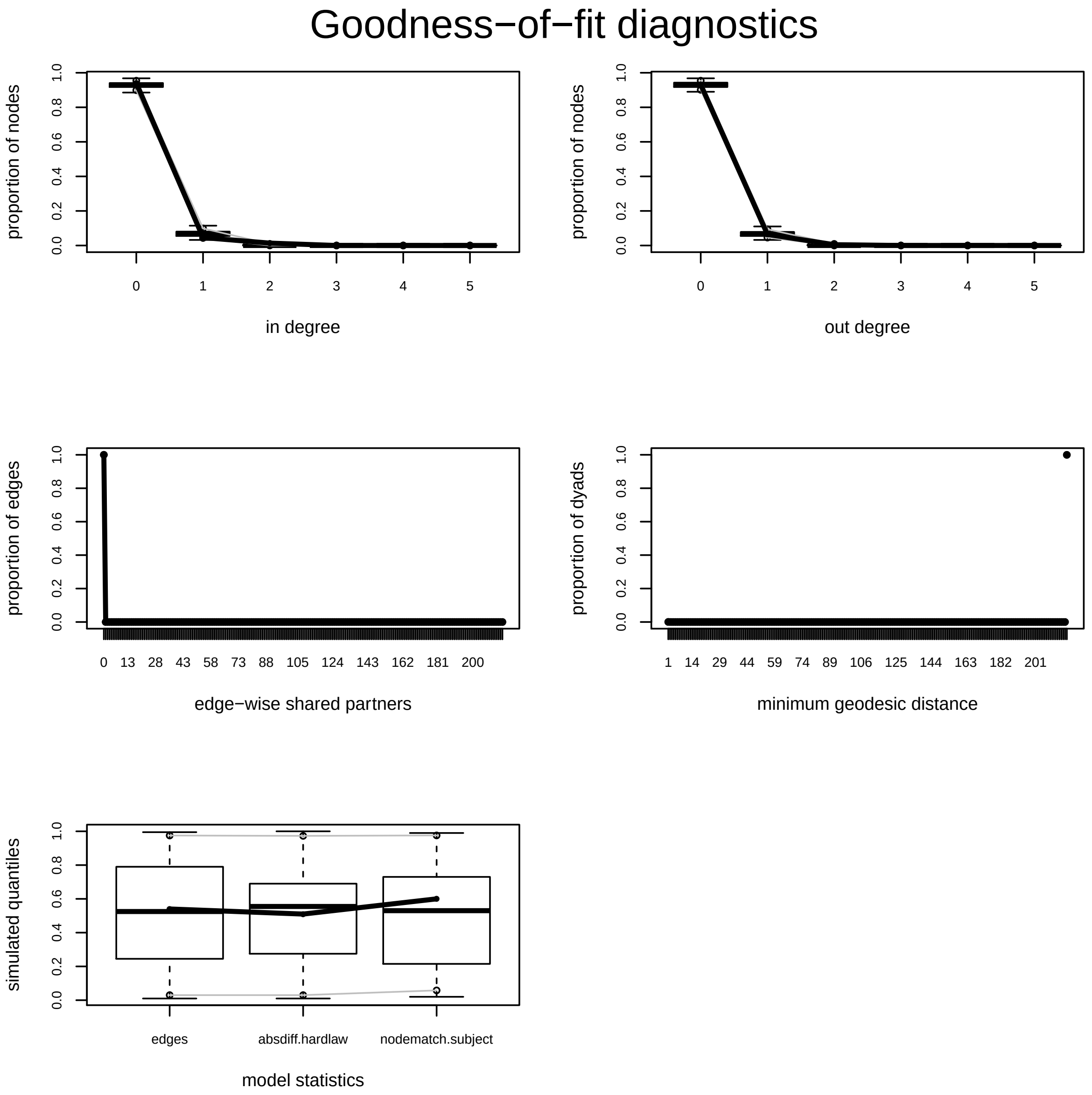

Figure 9: **Negative Treaty Influence ERGM Goodness of Fit Boxplots.** Boxes reflect networks simulated from model estimates, line reflects the observed statistics of the network. A well fitting ERGM should have observed statistics intersecting the boxplot medians. The Negative Treaty Influence Network ERGM, presented in Table 2 of the manuscript, appears to fit very well.

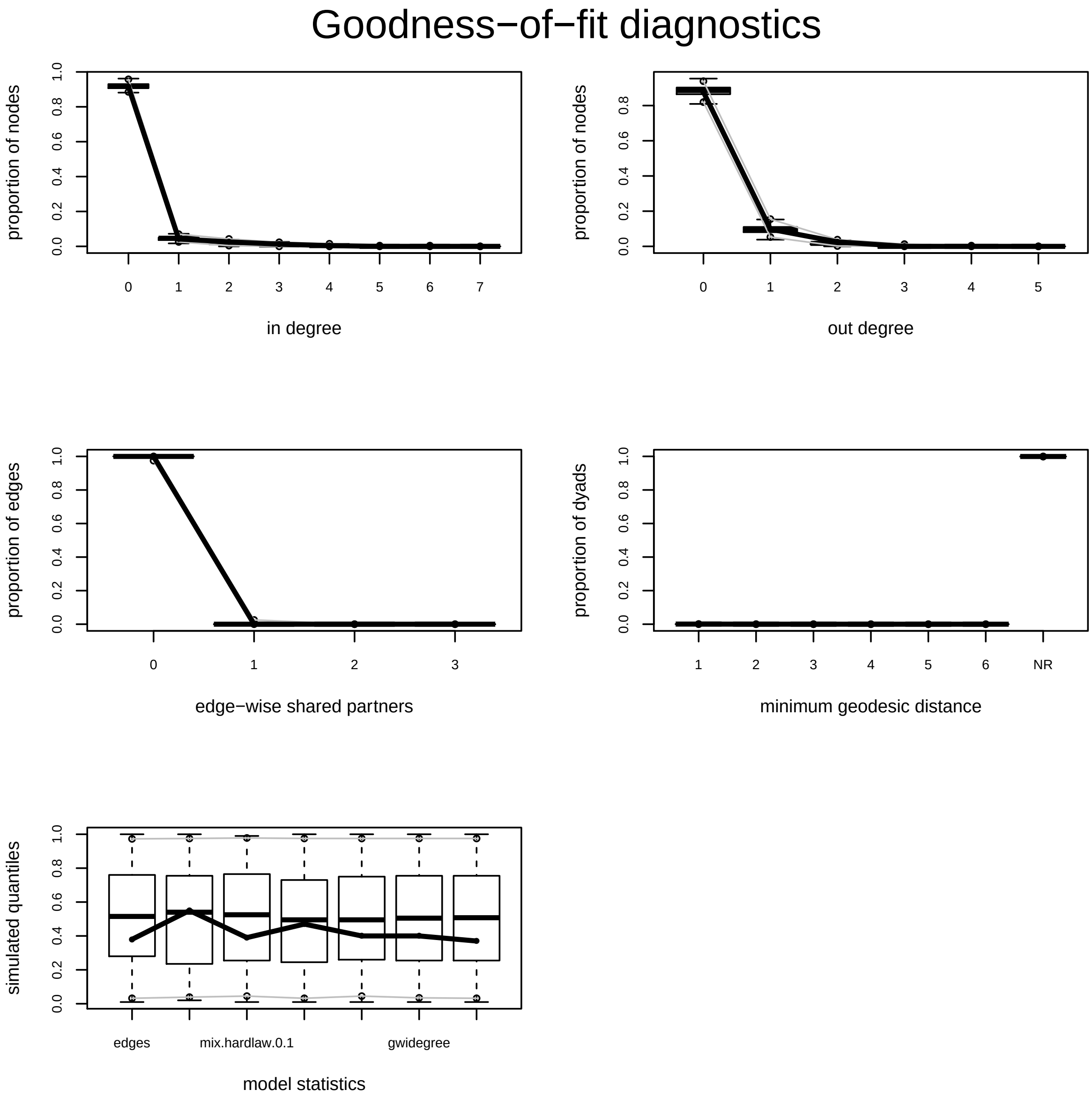

Figure 10: **Positive Treaty Influence ERGM Goodness of Fit Boxplots.** Boxes reflect networks simulated from model estimates, line reflects the observed statistics of the network. A well fitting ERGM should have observed statistics intersecting the boxplot medians. The ERGM for the Positive Treaty Influence Network, presented in Table 2 of the manuscript, fits the observed network well.

Accounting for European Coordination

Regionalization may lead to decreased transaction costs, decreased uncertainty, and increased information through providing institutionalized coordination to assist states in environmental treaty ratification (*14*). Not only may regionalization directly inform whether a country is more or less likely to ratify a treaty within a given year, it may lead to zones of ratification where states coordinate behavior through formal institutional organizations (IOs) to overcome collective action problems (*15*). These two motivations draw us to specify an additional BLIN model mirroring that of the manuscript, only differing in its addition of a dichotomous indicator variable for whether the state is a member-state of the European Union (EU) within the given year of an observation.

It would be unreasonable to assume that the addition of this indicator would not change the influence networks presented in the manuscript for two reasons. First, the new indicator should explain a great degree of residual variation from the initial model. This may lead to more confident estimates for the influence parameters and as such *actually* produce more significant entries in the $\mathbf{A}$ and $\mathbf{B}$ matrices. Second, we might expect that some of the influence parameters would be confounded by EU membership or non-membership, and as such, some of the entries significant in the $\mathbf{A}$ and $\mathbf{B}$ matrices presented in the manuscript would not be significant for this model.

Results roughly confirm this expectation – while the number of significant entries increases between models, the substantive size of entries is relatively constant. All predictor variables that were penalized to zero in the manuscript model were penalized to zero when including the EU indicator. In addition, all predictor variables – in this case only the global mean μ – remain significant when including the EU indicator, while the EU indicator is not significant when included. The network effects from the manuscript changed little when including the EU

indicator. The Jaccard scores for entries that are not penalized to zero were 0.988 and 0.978 for entries in $\mathbf{A}$ and $\mathbf{B}$, respectively, meaning that nearly every entry that was selected for the manuscript model was selected for the model when including the EU indicator and vice versa. Similarly, the point estimates of the $\mathbf{A}$ and $\mathbf{B}$ entries had R^2 values greater than 0.99 between entries that were not penalized to zero in either model. Lastly, when adding the EU indicator, 10.8% of entries in the $\mathbf{A}$ matrix and 10.6% of entries in the $\mathbf{B}$ matrix remain significant. The fact that the parameters not penalized to zero and their estimates change little when including the EU indicator, whereas the entries that are significant change in $\mathbf{A}$ and $\mathbf{B}$ indicates strong support for our proposition that confidence intervals are the only things that should change with the addition of the EU indicator.

We do not present the model that includes the EU indicator in the manuscript for three reasons. First, interpretation of the influence networks becomes much harder when you include other indicators solely designed to account for coordination or influence. It is substantively difficult to interpret what an influence relationship looks like when you strip away the effect of membership within particular regional-integration institutes. In other words, it may not make sense to explicitly consider the Franco-German influence relationship while stripping away an integral aspect of that relationship – joint membership in the EU. While it may give a cleaner read on what that parameter may look like, such a read would be an abstraction from reality. Second, it is unclear which regional institutes may or may not confound influence relationships. While it may seem reasonable to account for one or two institutions using indicators, the literature does not indicate a threshold for which organizations should or should not be included. While the inclusion of the European Union may seem obvious, the inclusion of the Organization of American States (OAS), North American Free Trade Agreement (NAFTA), Association of Southeast Asian Nations (ASEAN), and the Economic Community of West African States (ECOWAS) might seem reasonable as well. It is unclear where to draw the line between which

institutions should be included. Fully saturating the model with additional indicators for membership within a regional economic, cultural, or military institution would only serve to magnify the interpretation problems previously discussed and likely produce multicollinearity. Third, and finally, the model presented in the manuscript mirrors the state-of-the-art specification that has been used in the study of environmental treaties (*1*). Given that this model specification has been expertly vetted, we feel uncomfortable deviating too far. In addition, holding model specification constant allows for closer comparison of our results to the current state of the literature.

Treaty IDs and Names

Table 3 contains a table linking the Treaty IDs presented in the manuscript to their corresponding name.

Treaty ID	Treaty Name
2802	Convention For The Prevention Of Marine Pollution By Dumping From Ships And Aircraft
2832	Convention On The Prevention Of Marine Pollution From Land-Based Sources
2854	Protocol To The International Convention On Civil Liability For Oil Pollution Damage
3289	Convention On The Prior Informed Consent Procedure For Certain Hazardous Chemicals And Pesticides In International Trade
40440	International Convention for the Conservation of Atlantic Tunas
40452	International Convention for the Prevention of Pollution from Ships as modified by the Protocol of 1978
40477	Convention on the Prohibition of Military or any other Hostile Use of Environmental Modification Techniques
40505	Treaty on Principles Governing the Activities of States in the Exploration and Use of Outer Space Moon and other Celestial Bodies
40511	Convention on the Prohibition of the Development Production and Stockpiling of Bacteriological (Biological) and Toxin Weapons and on their Destruction
40549	Additional Protocol to the European Convention for the Protection of Animals during International Transport
40572	Convention on the Conservation of Migratory Species of Wild Animals
40601	Convention concerning the Protection of the World Cultural and Natural Heritage
40602	Convention on Wetlands of International Importance especially as Waterfowl Habitat
40606	Convention on Fishing and Conservation of the Living Resources in the Baltic Sea and the Belts
40615	Protocol for the Protection of the Mediterranean Sea against Pollution from Land-Based Sources
40659	International Convention on Standards of Training Certification and Watchkeeping for Seafarers
40687	International Tropical Timber Agreement (1984)
40710	Convention for the Protection of the Ozone Layer
40731	Protocol on Substances that Deplete the Ozone Layer
40793	United Nations Framework Convention on Climate Change
40794	Convention on Biological Diversity
40798	Agreement on the Conservation of Bats in Europe
40801	Convention on the Prohibition of the Development Production and their Destruction
40805	Regional Agreement on the transboundary movement of hazardous wastes
40808	Convention concerning Safety in the Use of Asbestos
40827	United Nations Convention To Combat Desertification In Those Countries Experiencing Serious Drought And/ Or Desertification, Particularly In Africa
40828	Convention on Nuclear Safety
40832	Energy Charter Treaty
40843	Energy Charter Protocol on Energy Efficiency and related Environmental Aspects
40869	Convention on the Prohibition of the Use Production and Transfer of Anti-Personnel Mines and on their Destruction
40873	Comprehensive Nuclear Test Ban Treaty

Table 3: **Treaty Identifiers and their Corresponding Names.** Rows show a given treaty ID number and its corresponding name.

References

1. G. Spilker, V. Koubi, The effects of treaty legality and domestic institutional hurdles on environmental treaty ratification. *International Environmental Agreements: Politics, Law and Economics* **16**, 223–238 (2016).

2. T. Bernauer, A. Kalbhenn, V. Koubi, G. Spilker, Is there a "depth versus participation" dilemma in international cooperation? *Review of International Organizations* **8**, 477-497 (2013b).

3. M. J. Marshall, K. Jaggers, *Polity IV Project: Political Regime Characteristics and Transitions, 1800-2014. Users' Manual* (College Park, MD: University of Maryland, 2015).

4. O. A. Hathaway, Treaties' end: The past. *Present, and Future of International Lawmaking in the United States* **117**, 1323–25 (2008).

5. T. Bernauer, T. Böhmelt, V. Koubi, Is there a democracy-civil society paradox in global environmental governance? *Global Environmental Politics* **13**, 88-107 (2013).

6. K. S. Gleditsch, Expanded trade and gdp data. *Journal of Conflict Resolution* **46**, 712-724 (2002).

7. J. Pevehouse, T. Nordstrom, K. Warnke, The correlates of war 2 international governmental organizations data version 2.0. *Conflict Management and Peace Science* **21**, 101–119 (2004).

8. D. B. Carter, C. S. Signorino, Back to the future: Modeling time dependency in binary data. *Political Analysis* **18**, 271-292 (2010).

9. R. Tibshirani, Regression shrinkage and selection via the lasso. *Journal of the Royal Statistical Society. Series B (Methodological)* pp. 267–288 (1996).

10. J. Friedman, T. Hastie, R. Tibshirani, Regularization paths for generalized linear models via coordinate descent. *Journal of statistical software* **33**, 1 (2010).

11. J. Taylor, R. Tibshirani, Post-selection inference for l1-penalized likelihood models. *Canadian Journal of Statistics* (2017).

12. J. D. Lee, D. L. Sun, Y. Sun, J. E. Taylor, *et al.*, Exact post-selection inference, with application to the lasso. *The Annals of Statistics* **44**, 907–927 (2016).

13. D. R. Hunter, S. M. Goodreau, M. S. Handcock, Goodness of fit of social network models. *Journal of the American Statistical Association* **103**, 248–258 (2008).

14. K. W. Abbott, D. Snidal, Why states act through formal international organizations. *Journal of conflict resolution* **42**, 3–32 (1998).

15. R. Axelrod, R. O. Keohane, Achieving cooperation under anarchy: Strategies and institutions. *World politics* **38**, 226–254 (1985).
